# Evolutionary mechanisms driving the evolution of a large polydnavirus gene family coding for protein tyrosine phosphatases

**DOI:** 10.1186/1471-2148-12-253

**Published:** 2012-12-27

**Authors:** Céline Serbielle, Stéphane Dupas, Elfie Perdereau, François Héricourt, Catherine Dupuy, Elisabeth Huguet, Jean-Michel Drezen

**Affiliations:** 1 Institut de Recherche sur la Biologie de l’Insecte, UMR CNRS 7261, Faculté des Sciences et Techniques, Université F. Rabelais, Parc de Grandmont, 37200, Tours, France; 2 IRD, Institut de Recherche pour le Développement, UR 072, Laboratoire Evolution, Génomes et Spéciation, UPR 9034, Centre National de la Recherche Scientifique (CNRS), 91198 Gif sur Yvette Cedex, France et Université Paris-Sud 11, 91405, Orsay Cedex, France; 3 Present address: Laboratoire de Biologie des Ligneux et des Grandes Cultures, UPRES EA 1207, Université d'Orléans, 45067, Orléans cedex, France; 4 Present address: INRA, USC1328, Arbres et Réponses aux Contraintes Hydriques et Environnementales (ARCHE), BP 6759, 45067, Orléans Cedex 2, France

**Keywords:** Polydnavirus, Bracovirus, Protein tyrosine phosphatase, Gene duplication, Positive selection

## Abstract

**Background:**

Gene duplications have been proposed to be the main mechanism involved in genome evolution and in acquisition of new functions. Polydnaviruses (PDVs), symbiotic viruses associated with parasitoid wasps, are ideal model systems to study mechanisms of gene duplications given that PDV genomes consist of virulence genes organized into multigene families. In these systems the viral genome is integrated in a wasp chromosome as a provirus and virus particles containing circular double-stranded DNA are injected into the parasitoids’ hosts and are essential for parasitism success. The viral virulence factors, organized in gene families, are required collectively to induce host immune suppression and developmental arrest. The gene family which encodes protein tyrosine phosphatases (PTPs) has undergone spectacular expansion in several PDV genomes with up to 42 genes.

**Results:**

Here, we present strong indications that PTP gene family expansion occurred via classical mechanisms: by duplication of large segments of the chromosomally integrated form of the virus sequences (segmental duplication), by tandem duplications within this form and by dispersed duplications. We also propose a novel duplication mechanism specific to PDVs that involves viral circle reintegration into the wasp genome. The PTP copies produced were shown to undergo conservative evolution along with episodes of adaptive evolution. In particular recently produced copies have undergone positive selection in sites most likely involved in defining substrate selectivity.

**Conclusion:**

The results provide evidence about the dynamic nature of polydnavirus proviral genomes. Classical and PDV-specific duplication mechanisms have been involved in the production of new gene copies. Selection pressures associated with antagonistic interactions with parasitized hosts have shaped these genes used to manipulate lepidopteran physiology with evidence for positive selection involved in adaptation to host targets.

## Background

Gene duplications have been recognized as an important source of evolutionary innovation and adaptation in a variety of organisms [1-5]. Here we study gene duplications in the genomes of a virus group, the polydnaviruses (PDVs), which are unique in their obligatory association with parasitoid wasps. We analyze a gene family which has been subjected to particularly strong expansion. The genes encode Protein Tyrosine Phosphatases (PTPs), well known in vertebrates for their role in regulation of signal transduction pathways.

PDVs are stably integrated as proviruses in the genome of their associated parasitoid wasps [6-8] and transmitted exclusively by chromosomal inheritance. Particle replication is restricted to wasp ovaries, and virus particles are injected into the lepidopteran host of the wasp during oviposition at the same time as wasp eggs. PDV particles enter host cells, but unlike pathogenic viruses they do not replicate in the infected cells. Instead they express a battery of genes that causes a series of host physiological disruptions including the suppression of the host immune defences allowing parasitoid larvae to develop successfully in an otherwise hostile environment [9-11]. This unique example of mutualism between a virus and a eukaryotic organism constitutes an evolutionary success in terms of species diversification, with tens of thousands of parasitoid species carrying PDVs.

Two genera of PDVs have been described. Bracoviruses (BVs) are associated with braconid wasps of the microgastroid complex, a monophyletic group comprised of six subfamilies (Adeliinae, Cardiochilinae, Cheloninae, Khoikhoiinae, Mendesellinae, Microgastrinae) [12]. Ichnoviruses (IVs) are associated with ichneumonid wasps of the subfamily Campopleginae and Banchinae.

PDV genomes packaged in the particles are comprised of multiple circular dsDNA molecules (or segments). For example *Cotesia congregata* Bracovirus (CcBV) possesses a genome of 35 dsDNA segments with a cumulative size of 739 kb [13,14]. The other unique feature of PDVs is that almost half of their genes belong to multigenic families (11 for CcBV) a rare feature within virus genomes [13,15-17]. These gene families encode potential virulence factors [13-19]. We hypothesized previously that the diversification of virulence genes into families reflects the adaptive pressures imposed on PDV genome evolution due to their role in parasitism success [20,21].

Using the age of fossils to calibrate the molecular clock, it was estimated that the braconid wasp ancestor of the microgastroid complex lived ≈100 Millions years ago (Mya). Wasps and their bracoviruses have diverged from a unique ancestral association following the integration of a nudivirus genome into that of this common ancestor wasp [22-24]. Since the integration, profound modifications have occurred. Indeed the nudivirus genome is no longer packaged but is used to produce particles that incorporate DNA encoding virulence factors essential for parasitism success [25].

Some of the virulence genes have been acquired by the packaged genome from the genome of the wasp at different time points of microgastroid diversification. Viral sugar transporter genes were, for example, recently shown to have a clear phylogenetic link with hymenopteran genes [6]. This strongly suggests that cellular copies of these genes have been transferred, via an unknown mechanism, to the proviral form of the PDV genome resulting in the incorporation of these genes into the particles. However, in most cases PDV packaged genes, including PTP genes, have diverged to such an extent that they are no more clearly related to insect than to vertebrate genes [20]. In addition, a few packaged genes have most probably been acquired from other viruses by lateral transfer [26,27].

The genes encoding protein tyrosine phosphatases (PTPs) are common to all sequenced bracoviruses except that of the wasp *Chelonus inanitus* (CiBV) which belongs to a basal group (Cheloninae) of the microgastroid complex, suggesting that PTP genes were acquired relatively early in the course of wasp-bracovirus evolution. PTP genes are not found in IVs, except in *Glypta fumiferana* Virus (GfV: a PDV associated with a wasp from the Banchinae subfamily and suggested to perhaps constitute a new PDV genus). PTPs of this virus form a distinct clade to BV PTPs, suggesting that PTPs of the two lineages either have a different origin or have evolved separately [17], although they do share a common structure (they consist of ≈300 amino acids corresponding to a single PTP domain). In all bracovirus genomes described, PTPs constitute by far the largest PDV gene family, with 27 members in *Cotesia congregata* bracovirus (CcBV), 33 members in *Cotesia vestalis* bracovirus (CvBV) [19], 13 members in *Microplitis demolitor* bracovirus (MdBV), 42 members in *Glyptapanteles indiensis* bracovirus (GiBV) and 32 in *Glyptapanteles flavicoxis* bracovirus (GfBV) [6,13-15]. This expansion is particularly striking in these relatively small virus genomes in comparison to the human genome that has only 107 PTP genes.

In vertebrates, PTP genes are known to play a key role in the control of signal transduction pathways by dephosphorylating tyrosine residues on regulatory proteins, with each PTP acting on a specific substrate [28]. Bracovirus PTPs show considerable diversity in their amino acid sequences, indicating that each one has the potential to interact with a different substrate [29]. Moreover PTP gene expression is regulated in a tissue-specific and time-dependent manner [29-33]. PDV PTPs are therefore likely to target signal transduction pathways in different cell types and involved in multiple physiological processes of the parasitized host. Interestingly, only a subset of these genes encodes catalytically functional PTPs [29,32,34,35]. However PTPs lacking phosphatase activity have been suggested to play a physiological role in trapping phosphorylated proteins in order to impair cellular PTP activity in a competitive way [29].

The precise biological functions of the different PTPs in host-parasitoid interactions are not known. However, certain mammalian bacterial pathogens such as the agent of the plague (*Yersinia pestis*) have been shown to inhibit phagocytosis by injecting PTPs, which disrupt the actin rearrangements occurring during filopodial extension [36]. PDV PTPs were therefore proposed to disrupt signalling pathways controlling hemocyte cytoskeleton dynamics, thereby inhibiting encapsulation. In accordance with this prediction, transient expression of MdBV PTP-H2 or PTP-H3 in *Drosophila* S2 cells led to a reduction of phagocytosis of *E. coli* by these cells [32].

Bracovirus PTPs have undergone a great extent of expansion in copy number that appears to have led to functional divergence and creation of important virulence factors [20]. It is thus particularly interesting to study how gene duplications occurred and how the duplicated genes have evolved in order to better understand their contribution to bracovirus evolution and plasticity. Therefore we used complete or partial bracovirus genome sequences to study the genomic organization and the transmission of duplications. In addition we measured the selection pressures operating on individual genes. These approaches enabled us to determine the molecular and evolutionary mechanisms at the origin of the expansion and diversification of the PTP gene family.

## Results

### PTP phylogeny

**Figure 1:**
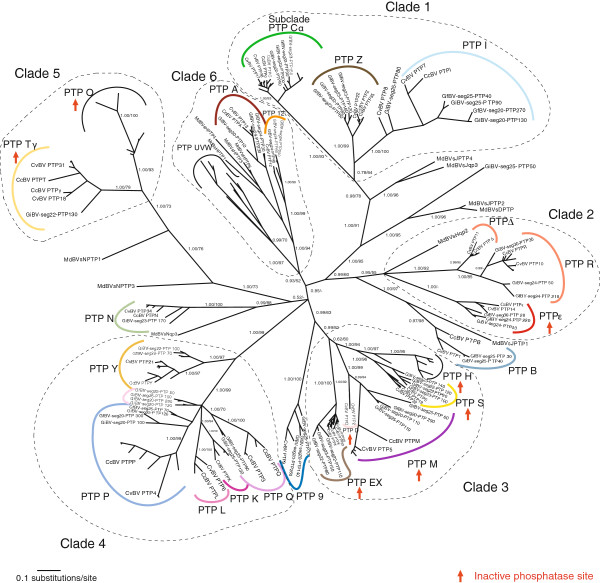
**PTP phylogenetic tree.** Unrooted PTP phylogenetic tree obtained from Bayesian inferences and Maximum Likelihood method. The numbers indicate the values of Bayesian posterior probabilities followed by the bootstraps values of Maximum Likelihood of the clades. Values below 50 are not indicated. Bootstrap values inside groups of orthologs are not shown for simplification. Sequence names are given in abbreviated form. For clarity only the names of the genes outside the main clades and those described in the analyses of duplications are indicated (the names of other genes can be visualised in Additional file 1). Color codes are those used in the duplications analyses (Figures 2 and 3, Additional files 3 and 4). PTP organization in clades 1-4 can be seen in detail in Figures 5 and 6.

Bayesian and maximum likelihood analyses of PTP sequences gave the same topology shown in Figure 1 (see also Additional file 1). We defined PTP subclades as the monophyletic copies, in different species, of a single PTP gene initially characterized in CcBV genome and named PTP A-Z and α−ε [29]. Most of the PTP subclades contain genes from several species, suggesting they originated before their speciation, others only contain copies from the same species suggesting they originated after the speciation event. For example certain subclades contain two weakly differentiated and virtually indistinguishable CcBV gene copies that appeared after speciation (Cα, EX, and Tγ subclades). In the case of R and Δ genes the copies appeared before speciation but they are still very similar in their sequences and were thus analyzed together in selection analyses. Almost all PTP subclades included *Cotesia* bracovirus, GiBV and GfBV sequences. These PTPs originated before the separation of the *Glyptapanteles* and *Cotesia* spp. Some subclades (PTP K, L) were specific to the *Cotesia* genus, they originated after the separation of *Glyptapanteles* and *Cotesia* spp and could therefore represent more recent gene copies. No orthologous MdBV genes could be identified, though several MdBV PTP sequences are in a basal position of some PTP clades (clades 1, 2 and 5, see Figure 1) or subclades (A, B and N) indicating a common history of most PTP lineages in all these species. This topology most probably reflects the basal position of *Microplitis demolitor* compared to the other species. Indeed, a previous study indicated that *Microplitis* species separated ≈53 mya, while the last common ancestor of the *Cotesia* and *Glyptapanteles* genera lived ≈17 mya [22].

### Comparison of bracoviruses associated with four wasp species reveals the history of segmental duplications and PTP gene acquisition and loss

**Figure 2:**
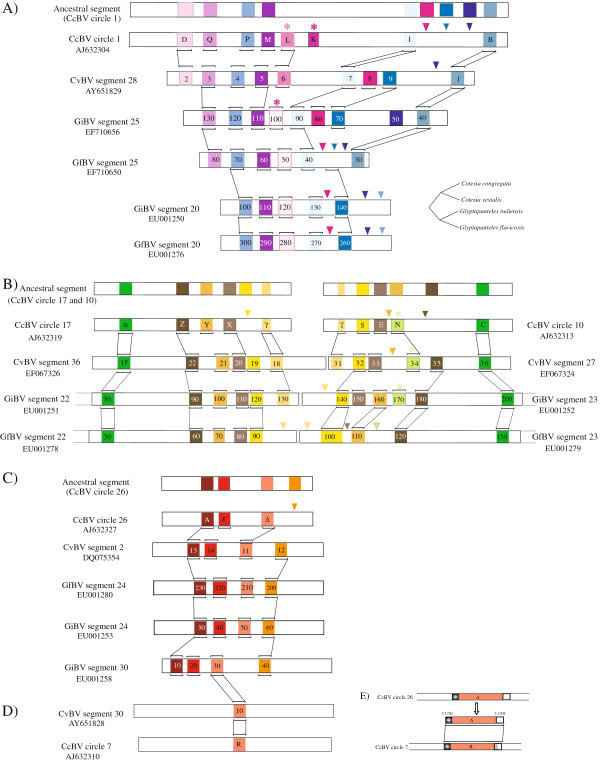
**Analysis of bracovirus segment PTP gene syntheny.** Orthologous and paralogous genomic regions from CcBV, CvBV, GiBV and GfBV are represented. PTP gene names correspond to those used in Genbank and the accession numbers of the segments are indicated. Phylogenetically related genes are indicated with the same colour. This color code is used throughout the publication. The lines indicate the block of homologous genes (**A**) Orthologous genes between CcBV circle 1 and related segments from CvBV, GiBV and GfBV, (**B**) Orthologous and paralogous genes between CcBV circle 17 and 10 and related segments, (**C**) Orthologous and paralogous genes between CcBV circle 26 and related segments. Stars indicate gene acquisitions and triangles gene losses. Proposed scenario of gene acquisition and loss in B (see the text for A and C): An ancestral segment was duplicated in mirror. Since this duplication the CcBV PTP N ortholog was lost specifically by GfBV. The homologues of CvBV PTP 35 and 19 were lost by CcBV, GiBV-seg23-PTP 160 and GfBV-seg23-PTP 110 orthologs were lost by both CcBV and CvBV (or acquired specifically in the *Glypatapanteles* lineage) and *Glyptapanteles* orthologs of CcBV PTP T was lost. (**D**) Isolated paralogous PTPs (PTP R) produced by dispersed duplications in CcBV circle 7 and CvBV segment 30. (**E**) Map of the regions of similarities between progenitor PTP Δ and the dispersed copy PTP R including 5’ and 3’ UTR sequences. The region in light grey corresponds to a stretch of non-homologous sequence.

In sequenced bracovirus genomes, PTP genes are clustered in particular segments, and the order of the different PTP genes is known [6,13,37] (see Additional file 2). By comparing the organisation of the genes in the different species and the monophyletic relationships deduced from the phylogenetic analysis (Figure 1) we found that the gene content and order of the different PTPs in *Cotesia* and *Glyptapanteles* species were mostly conserved. By comparison of the segments and phylogenetic relationships between the different genes (Figure 1) it was thus possible to retrace scenarios of PTP gene acquisition and loss (Figure 2 and Additional files 3 and 4), leading to the organisation observed today.

A) CcBV circle 1 and related segments: evidence of tandem duplications

In CcBV circle 1 and homologous segments, five PTP genes are conserved in the same order, namely PTP Q, P, M, I and B (Figure 2A). Each gene has an ortholog in CvBV segment 28 (genes 3, 4, 5, 7, 1), GiBV segment 25 (genes 130, 120, 110, 90 and 40) and GfBV segment 25 (genes 80, 70, 60, 40 and 30) respectively, suggesting that the different gene copies existed before the divergence between *Glyptapanteles* and *Cotesia* bracoviruses. In contrast, some genes are specific to a particular lineage (PTP D and L) or to a particular species (GiBV-seg25-PTP 50) or are conserved in most but not all segments (CvBV PTP 9).

We therefore hypothesize an evolutionary scenario explaining the differences between the orthologous segments by gene duplications and loss as described in Figure 2A: all the segments originated from a common ancestral form (inferred in Figure 2A) (a) the ancestor of CcBV PTP D and orthologous CvBV PTP 2 genes was probably lost by the *Glyptapanteles* lineage (indeed PTP D appeared before the separation of the two lineages according to Figure 1) (b) CcBV lost the orthologous genes of CvBV PTP 8 and 9 while PTP K and L were acquired in the Cotesia lineage and PTP K was lost specifically in *Cotesia vestalis* since this gene is found in *Cotesia* species more distant to *Cotesia congregata* (Additional file 1 and [38]) (c) PTP 50 (GiBV segment 25) present in GiBV only was most likely in the ancestor segment since the gene is ancient and belongs to a basal PTP clade comprising MdBV sequences (MdBVsJPTP2 and MdBVsDPTP).

Tandem duplications have occurred several times during the evolution of these segments, indeed Q, P, L and K genes are all closely related (Figure 1). By comparing the different segments and the ancestral form (Figure 2A), it can be deduced that Q and P have been produced before the separation of *Glyptapanteles* and *Cotesia* lineages, whereas L and K are only present in the *Cotesia* lineage (Figure 1).

B) CcBV circles 17 and 10 and related segments: evidence of mirror duplications

The CcBV circles 17 and 10 share paralogous PTPs (Figure 2B) and have orthologs in the other species. Indeed, CcBV circle 10 is a near replicate of circle 17, CvBV segment 36 is a near replicate of segment 27, GiBV segment 22 is a near replicate of segment 23 and GfBV segment 22 is a near replicate of segment 23. The chromosomal organization of these segments is known in the case of *Glyptapanteles* wasp species. The segments were shown to be associated “in mirror” in the proviral form of GiBV and GfBV as shown in the legend of Figure 2B [6] and were most probably produced by a mirror segmental duplication which occurred before the separation of *Cotesia* and *Glyptapanteles* lineages. From this hypothesis we can retrace gene gain and loss that occurred at different times during virus evolution as described in Figure 2B. Some of the events occurred in the genome of the common ancestor of *Cotesia* or *Glyptapanteles* lineages whereas others occurred more recently resulting in gene copy presence or absence in the segment of a single species. Interestingly, the duplications extend outside the viral segments in *G. indiensis* and *G. flavicoxis*, starting 941 bp upstream of segment 22 and finishing 902 bp downstream of segment 23. Furthermore, a 257 nucleotide spacer region corresponding to wasp sequences between the two viral segments is also duplicated indicating that the duplicated region was a chromosomal region including the segment, not the segment alone. This suggests that the duplication did not involve a viral mechanism.

C) Evidence of duplications involving whole segments dispersed in the wasp genome

Given the synteny of genes within CcBV circle 26 and CvBV segment 2 (Figure 2C) we can assume that they were inherited from a common ancestral segment. Only one copy of the segment is present in bracoviruses of *Cotesia* species while a duplication could be observed in GiBV segments 24 and 30, which share paralogous PTPs (Figure 2C). This strongly suggests that the duplication occurred in the genome of an ancestor of the *Glyptapanteles indiensis* lineage. Strikingly in this case, the two segments are not located at the same locus since the proviral form of segment 24 (isolated locus 4) is not adjacent to that of segment 30 (whose location is unknown) [6].

Similarly the pair of segments 20 and 25 (Figure 2A) probably originate from a duplication event that specifically occurred in the *Glyptapanteles* lineage since only one segment is present in *Cotesia* species. It is noteworthy that again segments 20 and 25 are not adjacent: segment 20 proviral form is located within the major bracovirus proviral locus comprising 66% of the segments, while that of segment 25 is isolated in the wasp genome. This organisation suggests that certain segments originate from reintegration of other segments, although this pattern could also be produced by genome rearrangement.

In certain cases, the occurrence of duplications can be suspected but the gene content of the segments has diverged in such a way that it is difficult to reconstruct the history of gene acquisition and loss. For example, PTP M, P and I from CcBV circle 1 are related to PTP X, Y and Z respectively from circle 17 (Additional file 4). The proviral forms of these segments are both isolated in the wasp genome, however it is unlikely that this common pattern was produced by the independent acquisition of individual genes. The duplication of an ancient segment in different locations of the wasp genome followed by divergence of the gene content appears to be a more likely explanation for this pattern.

**Figure 3:**
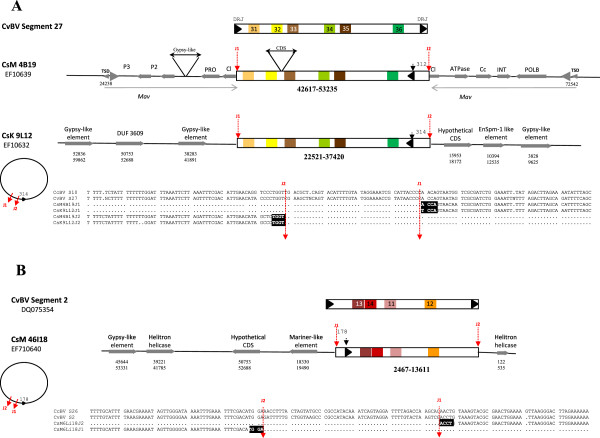
**A: Evidence for reintegration of a segment similar to CvBV S27 and CcBV Segment 10 in *C. sesamiae *Mombasa (CsM) and Kitale (CsK) strains.** CvBV S27: Expected proviral form of CvBV segment 27 flanked by Direct Repeat Junctions DRJ (black arrowheads). DRJ are involved in the production of the packaged circles from the proviral form. CsM 4B19: Genomic map of viral sequences inserted within a Maverick mobile element in CsM. Only one DRJ is present in the viral sequence indicating that the sequence does not correspond to the proviral form but probably to a virus circle reintegrated into the wasp genome (P3, P2, PRO, Cl, PolB, ATPase, Cc, INT, POLB: conserved genes of insect Maverick elements, Dupuy et al. (2011) [27]). CsK 9 L12: Genomic map of another segment highly similar to CvBV S27 and CcBV S10 reintegrated in a different genomic location, rich in gypsy-related retrotransposable elements in CsK. B: Evidence for reintegration of a segment homologous to CvBV S2 and CcBV S26 in CsM. Symbols are the same as in A. A and B: Alignments of the extremities of the reintegrated segments with homologous sequences of CcBV S10 and CvBV S27 (**A**) and CcBV S26 and CvBV S2 (**B**). The re-integrated segments have been cut at the same position during the integration process suggesting that circle reintegration involved a specific mechanism (see Figure 4). The red arrows indicate the position of the reintegration site relative to the DRJ on the circle. Junction sequences are indicated in bold.

**Figure 4:**
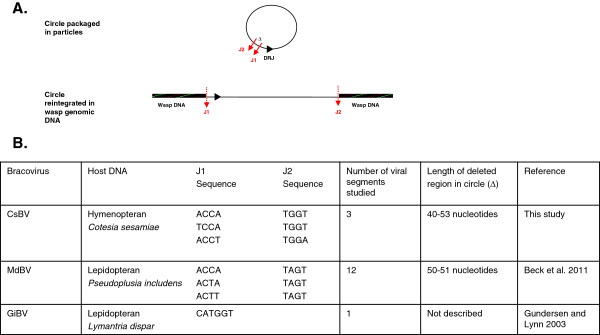
**Bracovirus circle reintegration in insect genomic DNA. A.** Proposed scenario of CsBV circle reintegration in *C. sesamiae* wasp genomic DNA. Reintegration of bracovirus circles occurs at viral boundary sites Junction1 (J1) and Junction2 (J2) and involves the deletion of a 40 to 53 nucleotides region of the circle (Δ). DRJ: direct repeat junction involved in virus excision. **B.** Bracovirus circle integration into lepidopteran genomic DNA has been shown for MdBV and GiBV. In MdBV J1 and J2 sequences are described as Host Integration Motifs and the integrated form has lost 50-51 nucleotides.

Strong evidence that PTP circle reintegration has indeed occurred in the wasp genome is given by the analysis of *Cotesia sesamiae* genomic sequences available in Genbank (see detailed analysis in Figure 3). Strikingly, segments homologous to CcBV circle 10 (CvBV S27) were found in two different genomic locations in *C. sesamiae* strains of Kenya. In *C. sesamiae* from Mombasa a sequence closely related to CcBV segment 10 is inserted within a Maverick transposable element [27] while in wasps from Kitale the same segment is inserted in a gypsy retrotransposon rich region (Figure 3A). A sequence highly similar to CcBV segment 26 (CvBV S2) was also found in *C. sesamiae* from Mombasa (Figure 3B). Sequence comparison of circular and reintegrated viral forms suggests that circle reintegration most probably involves a mechanism similar to the one described for the integration of bracovirus circles into lepidopteran host genomic DNA [39,40] (see Figures 3 and 4). Indeed, reintegration events have involved specific sites on the circle (J1 and J2 see comparison with MdBV host integration motifs in Figure 4) and have resulted in the deletion of a short 40 to 53 bp viral sequence (see Figure 4).

Altogether the identification of sequences from the same segment inserted in different locations of *Cotesia sesamiae* genome and the localisation of paralogous packaged segments in different regions of the genomes of *Glyptapanteles* species strongly suggest that segment reintegration events have played an important role in PTP gene expansion*.*

D) Evidence of a dispersed duplication

Although most of the PTP gene family expansion can be explained by duplications of entire segments, some examples of dispersed duplications involving a single gene could also be found. Indeed CcBV PTP R located on circle 7 is closely related to PTP Δ on circle 26. The region of similarity between these segments was detected by blastn analysis and extends from 80 nucleotides upstream of the translation start of PTP Δ to the entire coding sequence (with 71% similarity between PTP Δ and PTP R). A similarity is also detected 3’ of the coding sequence separated by a stretch of non-homologous sequence (see Figure 2E). The upstream sequence might correspond to a mRNA leader sequence (5’ UTR) because GiBV PTP gene transcription start sites have been mapped to regions comprising 30 or 112 nucleotides upstream of the translation start codon in segment 25 [30]. The observed dispersed duplication does therefore not encompass the gene promoter. Since the segments do not share other similar genes or other similar regions, this pattern suggests that PTP R has been produced through a dispersed duplication involving the reverse transcriptase-mediated insertion of a PTP Δ cDNA (more basal in the phylogenetic tree than PTP R, see Figure 1) or a paralogous copy closer to GiBV-seg30-PTP 30 within an ancestral form of circle 7 (Figure 2C). The loss of an intron would provide proof of such a mechanism, however, there are no introns in PTPs. Similar analyses indicate that dispersed duplications were probably involved in the production of PTP N from a cDNA corresponding to an ancestral GfBV-seg27-PTP 10 and in the production of GiBV-seg28-PTP 10 from an ancestor of GfBV-seg22-PTP 30 (data not shown).

In conclusion, duplications appear to be a major molecular mechanism involved in PTP diversification. They have occurred at different times during the evolution of the Microgastrinae lineage and were also accompanied by gene loss. We found evidence for the occurrence of classically described duplications (segmental, tandem and dispersed) as well as indications that uncommon duplications have occurred leading to similar PTP segments located in different parts of the wasp genome.

### Episodes of positive selection during PTP evolution

**Figure 5:**
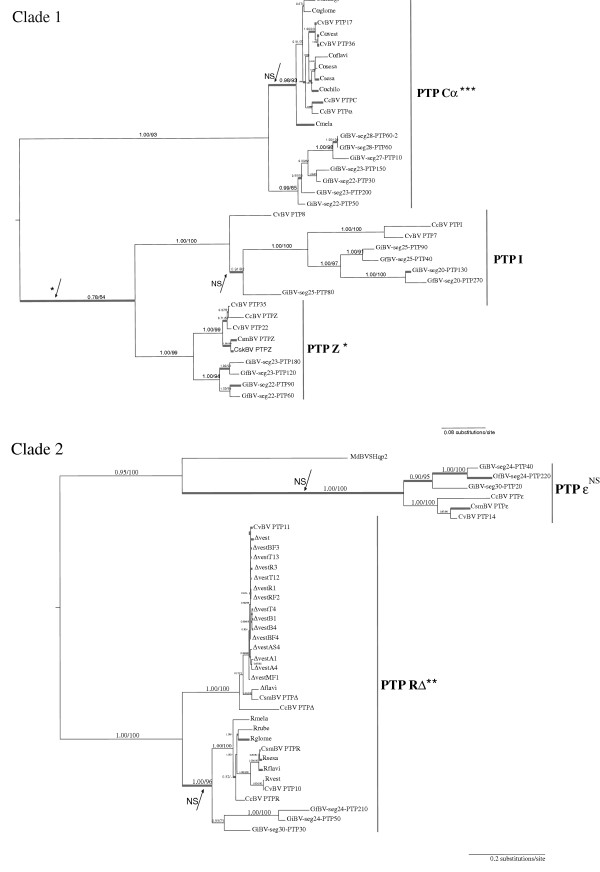
**Selection analysis of PTP evolution (Clades 1 and 2).** Unrooted phylogenetic trees were obtained from Bayesian inferences under the GTR + I + G substitution model. Posterior probabilities and bootstraps are indicated on the left of clade branches. Thick branches indicate ω>1 estimated under the branch-specific model in PAML. Some particular branches (indicated by arrows) or group of branches of a same clade with ω>1 have been tested by MA/MAnull model comparisons; among the branches tested stars indicate those under significant selection and NS those not significantly under positive selection (see Table 2). Although defined as different subclades PTP R and Δ have been analyzed together.

**Figure 6:**
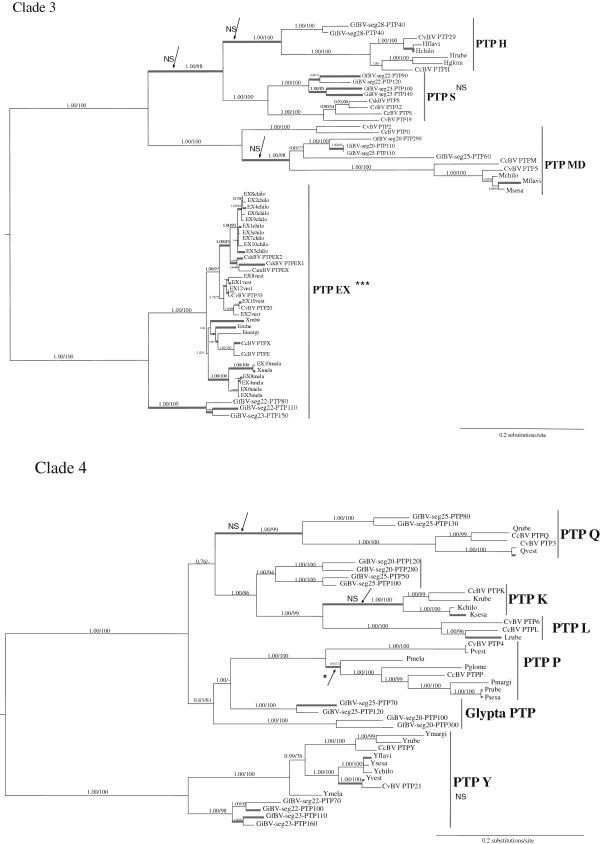
**Selection analysis of PTP evolution (Clades 3 and 4).** Unrooted phylogenetic trees from Bayesian inferences under the GTR+ I + G substitution model of clade 3 and clade 4 (see Table 2). Symbols are the same as in Figure 5. Although defined as different subclades PTP D and M have been analyzed together because their sequences are very close.

To study selection pressures that acted on PTP gene evolution we measured branch specific selection in four PTP clades independently (clades 1 to 4). Each clade was shown to diverge under varying selection pressures depending on the branch (Figures 5 and 6). Indeed the model that allows branches to evolve under different selective pressures (M0b) better explains the evolution of the PTP gene family compared to a model with no branch specific selection (M0) (see Table 1 for comparison of models by LRT). Branches (in Figure 5 and 6) that displayed positive selection (ω > 1) were tested for significance by MA/MAnull model comparisons (Table 2). A certain number of these branches were in fact shown not to be under significant positive selection (NS) or could not be tested due to insufficient numbers of sequences. Branches that displayed significant positive selection are indicated by a star (Figure 5 and 6). In addition subclades where many branches displayed positive selection (RΔ, Cα, EX, Z, ε, S, Y) were tested by LRT, and those under significant positive selection are indicated by stars and discussed here.

**Table 1:** Model comparisons and position of positively selected sites

**Cluster**	**LRT**		**Sites selected (site number)**
	**M8/M8a**	**M0b/M0**	
**PQLKY**	**NS**	**P=0.016**	
P	P=0.05	P<10^-3^	
Q	P=0.038	NS	
KL	NS	NS	
Y	NS	NS	
**DMHSEX**	**NS**	**P<10**^**-3**^	
MD	NS	P<10^-3^	
S	P=0.04	P<10^-3^	
H	NS	P<10^-3^	
EX	P<10^-3^	NS	9 sites
**RΔε**	**NS**	**P=0.002**	
RΔ	NS	P=0.004	
**ε**	P=0.03	P<10^-3^	1 site
**CαIZ**	**P=0.03**	**P<10**^**-3**^	
Cα	NS	P=0.048	
Z	P=0.002	P=0.03	

*Model comparisons were performed using Likelihood Ratio Test (LRT) between nested models.

Model descriptions: Branch selection models: M0=one class of ω ratio and M0b=tree different ω ratio for the branches. Site selection models (ω ratio varies according to two classes); M8a: beta distribution of ω and ω=1 and M8: beta distribution of ω and ω_1_ > 1.

**Table 2:** Branches or group of branches tested by branch selection models

**Hypothesis**	**Test MA/MAnull**	**Likelihood values**	**χ2 value**	**df**	***LRT p*-value**	
**Clade 1**						
Clade Cα	MA	7035.70	27.77	1	1.36E-07	<0.001
	MAnull	7049.58				
Branch Cα	MA	7058.20	0	1	1	NS
	MAnull	7058.20				
Branch I	MA	7058.08	0	1	1	NS
	MAnull	7058.08				
Clade Z	MA	7050.24	5.25	1	0.022	<0.05
	MAnull	7052.87				
Branch IZ	MA	7055.64	3.58	1	0.049	<0.05
	MAnull	7057.58				
**Clade 2**						
Clade ε	MA	2591.88	0	1	1	NS
	MAnull	2593.91				
Branch ε	MA	2601.60	0	1	1	NS
	MAnull	2601.60				
Clade RΔ	MA	2026.57	7.45	1	0.006	<0.01
	MAnull	2030.29				
Branch RΔ	MA	2601.78	0.03	1	0.86	NS
	MAnull	2601.80				
**Clade 3**						
Clade S	MA	5205.54	3.15	1	0.076	NS
	MAnull	5207.11				
Branch H	MA	5211.87	0.05	1	0.816	NS
	MAnull	5211.90				
Branch HS	MA	5211.93	0	1	1	NS
	MAnull	5211.93				
Branch M	MA	5211.93	0	1	1	NS
	MAnull	5211.93				
Clade EX	MA	5193.81	13.47	1	2.42E-04	<0.001
	MAnull	5200.55				
**Clade 4**						
Branch Q	MA	6960.59	0	1	1	NS
	MAnull	6960.59				
Branch K	MA	6956.33	0	1	1	NS
	MAnull	6956.33				
Branch P	MA	6958.79	5.14	1	0.023	<0.05
	MAnull	6961.36				
Clade Y	MA	6960.24	2.75	1	0.097	NS
	MAnull	6961.61				

*Model comparisons were performed using Likelihood Ratio Test (LRT) between nested models.

Model MA defines four classes of sites, where the two last classes have ω > 1 on the lineage of interest and ω < 1 for the rest of branches. This model is compared with MAnull which imposes ω=1 for the latter two classes.

In clade 1, several branches were shown to evolve under positive selection, with notably 35% of branches evolving under significant positive selection (ω > 1) both in the PTP Cα and Z subclades (p < <0.05 and p < 0.05, respectively) (Table 3). Remarkably, CcBV PTP C and α resulting from a segmental duplication evolved under opposite selective forces: positive versus negative (Figure 5, and refer to Figure 2B for segmental duplications). In clade 2, we observed a significant episode of positive selection between species within the PTP RΔ subclade (p < <0.05) (Figure 5). In clade 3, branches at the origin of the PTP S and EX subclades underwent purifying selection and a significant episode of positive selection is visible between and within species of the PTP EX clade (p < <0.05) with more than 47% of branches with ω>1 (Figure 6, Table 3). Again CcBV PTP X and E, produced by the same segmental duplication as Cα, have been submitted to contrasting selection pressures. CcBV PTP X has undergone positive selection, whereas CcBV PTP E has evolved under purifying selection. A similar pattern of opposite evolution of duplicated genes is observed for GiBV-seg22-PTP110 and GiBV-seg23-PTP150 (Figure 6, and refer to Figure 2B). Finally, evolution of PTP genes in clade 4 is better explained by purifying or nearly neutral selection since at least 85% of branches for each PTP genes present ω < 1 (Table 3, Figure 6). Certain ancestral branches appear to be positively selected, but only the value of the *Cotesia* PTP P branch is significant (p < 0.05).

**Table 3:** Percentage of branch length for five classes of ω ratio for the different genes

**PTP genes**	**% of clade branch length with :**				
	**ω<0.2**	**0.2<ω<0.5**	**0.5<ω<1**	**1<ω<2**	**ω>2**
Ι	6.6%	34.4%	50.7%	3.3%	5.1%
Ζ	17.5%	1.7%	45.6%	10.3%	24.9%
Cα	3.0%	26.3%	42.5%	21.5%	6.8%
RΔ	70.3%	16%	9.7%	0%	4.0%
ε	0%	0.4%	0.2%	0.3%	10,10%
MD	34.2%	16.2%	45.1%	4.5%	0%
H	56.7%	31.1%	8.1%	3.8%	0.3%
S	24.6%	21.1%	15.6%	19.1%	19.7%
EX	1.2%	30.6%	20.8%	28.0%	19.4%
P	3.6%	51.9%	30%	12,50%	2%
Q	7.1%	75.6%	14.2%	0%	3,10%
KL	0.8%	48.2%	39.1%	11.9%	0%
Y	25.2%	35.9%	27.1%	8.2%	3.6%

The ω ratio for each branch was estimated using branch specific codon substitution models in PAML.

These analyses show that recent episodes of positive selection have occurred in certain PTP subclades. It is noteworthy that many of the gene copies involved originated from the mirror segmental duplication (C, α, Ε, X, Z) and the dispersed duplication (R,Δ). In contrast to other PTPs which are relatively fixed, these PTPs have undergone a recent evolutionary burst which resulted in new PTP alleles or copies, and in several cases the evolution of these PTPs follow models of duplication that involve positive selection of one of the duplicated gene copies [41].

### Bracovirus PTP structure

**Figure 7:**
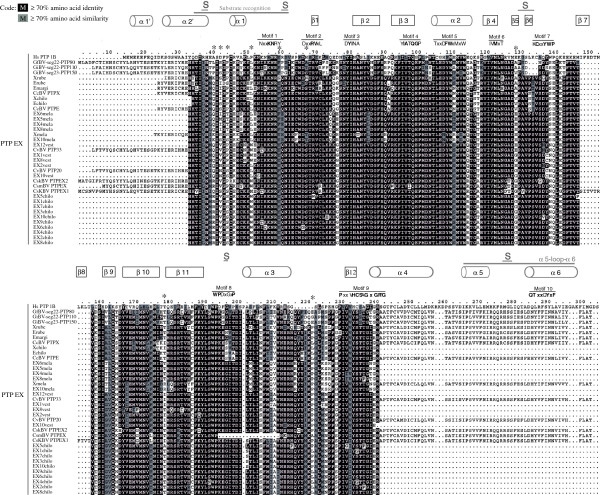
**Positively selected sites in PTP EX subclade.** In the alignment, PTP EX sequences are represented with the same amino acid numbering to facilitate comparisons. Structural motifs identified in Vertebrate PTPs are indicated. Variable regions α1/β1 loop, α5-loop-α6 and sites involved in substrate specificity (noted S) are mentioned. Stars indicate sites significantly shown to evolve under positive selection.

Before discussing the sites involved in positive selection, it is important to consider the structure of bracovirus PTPs based on crystallographic, computer modelling (Cα- regiovariation score analysis) and mutant studies performed on vertebrate PTPs. Usually in cellular PTPs, the PTP domain is associated with other conserved protein domains involved in modulating the function of the protein. However bracovirus PTPs consist essentially of a protein tyrosine phosphatase domain, like PTP1B, one of the best characterized vertebrate PTPs. Bracovirus PTP sequences carry the 10 conserved motifs that characterize the protein tyrosine phosphatase domain defined in vertebrate PTPs [29] (Figure 7). Two kinds of motifs can be described from crystallographic analyses and mutant studies [28], the structural motifs affecting PTP secondary or tertiary structure (motifs 2, 3, 4, 5, 6, 7), and those directly involved in phosphotyrosine recognition and phosphatase activity (motifs 1, 8, 9 and 10). Conservation of these motifs in bracovirus PTPs differs depending on the PTP clades considered.

In clade 3 (Figure 1), clade TγO and subclade PTP ε (see Additional file 5) a major functional difference occurs in motif 9 where the cysteine residue that is essential in the catalytic site for the PTP activity is mutated to glycine or serine. This results in proteins that have lost phosphatase activity as shown experimentally in the case of CcBV PTP M [29]. Mutations of the cysteine to serine or alanine have been shown to abrogate all enzyme activity while maintaining affinity for substrates *in vitro*, a feature that is used to obtain PTPs in complex with phosphotyrosine substrates (mutant trapping) [28]. All bracovirus PTP EX proteins harbour a serine (or a glycine) instead of a cysteine suggesting these enzymes function as traps for tyrosine phosphorylated proteins. Furthermore, PTP EX proteins are also mutated in the catalytic site surface loop (motif 8), notably at the level of the aspartic acid, that normally plays the role of a catalyst. Again PTPs with substitutions of aspartic acid for other amino acids form stable complexes with phosphorylated substrates, strongly supporting the hypothesis that PTP EX may act by trapping phosphorylated proteins.

All the other PTPs have preserved motif 9 [29] carrying the catalytic site suggesting that although these proteins are very divergent and eroded in other conserved motifs they may have retained a phosphatase activity. Indeed CcBV PTP A and MdBV PTP-H2 were shown to be functional phosphatases [29,35].

### Positive selection acted on specific residues

To determine whether the positive selection observed acted on specific amino acid residues, we measured site selection in the subclades of PTP clades 1 to 4 using the site selection model (M8), as shown in Table 1.

PTP EX and PTP ε clades were better explained by the M8 selective model and significant positively selected sites could be identified in 9 sites and 1 site, respectively (Table 1). In PTP ε the positively selected site is situated after motif 10, in a region not described as involved in determining target specificity (Additional file 5). The position of the 9 sites under positive selection in PTP EX, are indicated in Figure 7. Five of these sites are situated in regions that are involved in defining substrate selectivity in human PTPs (http://ptp.cshl.edu &http://science.novonordisk.com/ptp) [28]. Although none of these sites has yet been characterized by mutational studies, one site in the very same region in human PTP1B, arginine (24) (R position 37 in our alignment, see Figure 7), was shown to be engaged in the second phosphotyrosine binding site of this protein [42-44]. The concentration of sites under positive selection in this particular region of PTP EX proteins involved in determining substrate specificity is unlikely to be expected by chance, and could correspond to an ongoing adaptation of these PTPs to a new target.

Another site that we identified as positively selected in PTP EX and that is very likely to influence PTP interaction with its substrate, is situated at the level of the normally conserved glutamic acid (E) in position E115 in human PTP1B (position 128 in our alignment, see Figure 7). In PTP1B, glutamic acid 115 was identified along with another residue to form hydrogen bonds with the PTP loop, and is therefore involved in defining the architecture and function of the phosphate-binding loop [28]. It is conceivable that modifications of this architecture may induce variations in the type of substrates these PTP EX are capable of trapping, and may therefore directly influence PTP EX substrate specificity and affinity.

## Discussion

The association between endoparasitic wasps and viruses is the only mutualism involving an eukaryote and a virus identified so far. The virus particles produced in wasp ovaries are used as a tool to deliver genes –most probably mainly of wasp origin- that are expressed by the parasitized host to ensure parasitism success. Our study reflects the dynamic evolution of the PTP genes integrated into the wasp genome and present in the DNA packaged in the particles delivered to the host. They have undergone multiple gene duplication events and several episodes of natural selection. This large expansion -unique for a viral genome- is most probably related to their role in wasp parasitism success. Gene duplications are likely to have offered new sources of innovation allowing wasps to colonize new hosts or to maintain parasitism success in the changing physiological environment of hosts adapting their defences to these parasites (host-parasitoid arms race).

The PTP gene family is the most diversified family found in polydnaviruses associated with braconid wasps [13,15,16,19]. Our study focused essentially on PTP genes from bracoviruses associated with *Cotesia* and *Glyptapanteles* species giving an overview of ≈17 million years of bracovirus PTPs evolution [22]*.* These genes are annotated in several PDV genomes, offering support to gain insights on the molecular mechanisms, which produced this large gene family. Bracovirus PTPs are therefore a particularly good example to study how duplications are produced and maintained in the context of host-parasitoid relations.

### PTP duplication mechanisms

Four major mechanisms appear to be involved in PTP diversification; segments have been duplicated (i) by segmental duplication or (ii) by reintegration in the wasp genome, individual genes have been duplicated (iii) in tandem or (iv) dispersed.

(i) Segmental duplications are clearly involved in PTP diversification. Indeed CcBV circles 10 and 17 are contiguous in the wasp genome [6]; Bezier A, unpublished data] and harbour five homologous PTP genes suggesting they arose from a segmental duplication. These processes have previously been proposed to play a critical role in primate evolution in creating new genes and shaping human genetic variation [45]. They seem particularly important in stimulating evolutionary changes since five recently positively selected bracovirus PTPs emerged from the same segmental duplication (e.g. PTP E and X, PTP C and α, and PTP Z; see Figure 2B).

(ii) Several duplicated segments are located at different positions in the wasp genome such as the segment pairs 24/30 and 20/25 of the *Glyptapanteles* species. PTP-containing segments (10, 17 and 26) have also been shown to be reintegrated in isolated positions of *C. sesamiae* genomes. Although it cannot be completely excluded that they have been produced by segmental duplications and later separated by chromosomal rearrangements or insertions of mobile elements over time, this pattern might have been produced by a mechanism that is specific for bracoviruses (Figure 4). Indeed GiBV segment 25 (also known as segment F) was shown to integrate into the genome of cultured insect cells [46] and parasitized host DNA [47] and thus behaves like a mobile element. Beck *et al.*[39] also showed very recently that two MdBV segments have the capacity to integrate and persist in infected lepidopteran host DNA [39]. Our viral boundary sites of reintegration (junction regions) are not strictly conserved but resemble those described in GiBV (for 1 segment) and MdBV (for 12 segments, host integration motifs), and moreover a stretch of viral sequence is lost during the process of integration suggesting that bracoviruses use common mechanisms to reintegrate into genomic (wasp or lepidopteran) DNA [39,40] (Figure 4). Parasitoid wasp eggs and larvae are bathed in the haemolymph that contains PDV particles, which have the ability to enter a wide range of cell types of the lepidopteran host. It is thus conceivable that some segments could enter into the wasp germline and integrate into the genome, producing new functional segments in some rare cases, provided that they could be amplified and excised during virus particle production. The direct repeats (DRJ) that normally flank the integrated form of the virus segments [48] (also called wasp integration motifs, WIM, in [39]) and that are thought to be required for packaging of segments in virus particles are lacking from CsBV reintegrated circles. Only one element of the repeats remains, indicating that these sequences most likely do not produce packaged circles. However it is conceivable that duplication of such reintegrated forms could fortuitously restore a sequence with two direct repeats and thus could create a newly packaged segment.

Notably, all the *G. flavicoxis, G. indiensis* and *C. congregata* PTP containing segments are dispersed (not associated with a macrolocus) in the wasp genome (except for segment 20 of the *Glyptapanteles* lineage) and all dispersed proviral loci encode PTPs (Additional file 2). A specific ability of PTP segments to integrate into DNA might explain this feature: most of the different PTP-containing segments are dispersed in the wasp genome and may originate from rare reintegration events that occurred successively during wasp evolution. Accordingly the three identified cases of reintegration involve PTP containing circles. This phenomenon might have played a major role in the expansion of the PTP gene family.

(iii) Tandem duplications of genes are thought to be the major mechanism for the creation of new genes and this process has been documented in several organisms [49-52]. In bracoviruses this pattern was observed for PTP K, L, P, Q genes found in CcBV circle 1. Based on the PTP phylogeny, we can suggest that these genes were produced after several rounds of duplications, which occurred at different periods. These results emphasize that PTP tandem duplications constitute a dynamic lineage specific process.

(iv) Several examples suggesting the occurrence of dispersed duplications of individual genes could be found among PTP genes. This process is thought to be mediated by reverse transcriptases of endogenous retrotransposons [53] that are likely to be present in parasitoid genomes since several remnants of retroelements were detected in CcBV [26]. This mechanism produces intronless genes that have lost their original promoter. The fact that the duplicated region, in circle 7, includes 5’ and 3’ UTRs is a strong argument that PTP Δ cDNA was duplicated by retrotranscription and reinserted giving rise to PTP R. This mechanism of gene acquisition was already proposed for CcBV cystatin genes which do not contain introns in contrast to cellular cystatin genes [54]. Only genes expressed in the germ line can be duplicated via this process and this is likely to be the case as PTP genes have been shown to be expressed in wasps [33,55].

By comparison of homologous segments, we could also identify evidence for gene loss implying that PTP gene evolution matches the “Birth and Death” model described by Nei and colleagues [56]. According to this model, genes arise continuously by duplication and are lost by deletion or by mutational events. An ongoing process of pseudogenization was also observed for copies corresponding to different PTP genes in different species [29] (Additional file 5). Thus, some PTP gene copies were lost while others were created by duplications and transmitted in particular lineages. As it has been shown in primates or in *Drosophila*, gene expansion and contraction could explain important adaptive traits allowing physiological adaptations of their host species [57,58]. By studying bracovirus PTP genes, we showed that genome reorganisation occurred on a very fine evolutionary scale with gene acquisition and loss occurring between species. Braconid wasps associated with polydnaviruses have been shown to be a highly diversified group composed of species with a very narrow host range [59] and virus genome plasticity could be viewed as a powerful mechanisms allowing wasp adaptive radiation. Indeed PTP gene expansion may be a source of evolutionary innovations offering wasps dynamic adaptive viruses.

### How did PTP family divergence occur?

Understanding evolutionary processes underlying fixation of duplications and divergence of duplicated copies is of major interest to determine how genes can be created and how new functions could appear. For classical models, duplications do not affect fitness and the fixation of the duplicated copy is a neutral process [60-62]. In contrast, in selective models, duplications are immediately advantageous either by resulting in a beneficial increase in the amount of protein produced, or by providing the immediate opportunity for the emergence of a new function [41,63]. The results obtained on certain bracovirus PTPs, such as PTP EX, showing that positive selection has been involved in bracovirus PTP copy divergence, sustain the second class of models. We detected selected amino acids within the PTP EX cluster which has undergone a recent evolutionary burst. Most of these residues were shown to occur in regions predicted to be involved in PTP substrate specificity suggesting a recent shift in target of these PTPs.

Furthermore, the fact that an excess of alleles can be observed for certain PTP EX (7 alleles were isolated from two *Cotesia chilonis* wasps) could be an indication that certain PTPs follow an adaptive radiation model of evolution [64]. In this model, gene alleles in the population are partially adapted to perform a new function but not efficiently. In the case of parasitoid wasp host shift, the partially adapted protein might be a PTP already fitted to a lepidopteran target (from the former host) having to interact with the homologous molecule of another lepidopteran species (the new host). In the context of a parasitoid-host arms race a partially adapted PTP may enable the parasitoid to circumvent lepidopteran host resistance. According to the adaptive radiation model, new copies are produced by successive rounds of duplications, to compensate for the low affinity of the partially adapted protein by dosage effects. The effect of different mutations can be assessed, until a protein with increased affinity for the new target is produced. Duplications of partially adaptive alleles, followed by positive selection, can lead to genes carrying a full function. Once such a goal is achieved, the other copies become pseudogenes and are eventually lost [64].

To date there is only partial evidence that bracovirus PTP evolution has resulted in genes with different targets and functions. Some bracovirus PTP genes play an important role in host immune alteration particularly by modulating PTP cell activity in hemocytes [32]. They were also suggested to be involved in controlling larval development by acting on the level of phosphorylation of regulatory proteins involved in the prothoracic gland’s response to prothoracicotropic hormone (PTTH) required to produce ecdysteroids [65]. One particular class of PTPs has been identified for not carrying PTP activity, but the proteins were shown to reduce PTP cell activity probably through competition with host PTPs [32,34]. Furthermore, some PTPs are differentially expressed in the course of parasitism suggesting they perform different functions [30-33,35]. PTPs can also play a role in host behaviour manipulation. A PTP expressed by a baculovirus was shown to enhance locomotory activity so that the infected larva climb to the top of the plants to release particles and thus increase baculovirus transmission [66]. Altogether, these data emphasize the potential for high functional diversity in bracovirus PTPs, with PTPs involved in immunity, and possibly development and behaviour that are processes that are all targeted during parasitism. However the determination of the target and precise role of each PTP is needed for a detailed understanding of the role of functional innovations in bracovirus PTP evolution.

The challenge is thus now to study the role and regulation of PTPs in relation with the mutational events that occurred after duplications.

## Conclusions

This study is the first detailed description of the mechanisms involved in the expansion of polydnavirus genes within viral and insect genomes. Most polydnavirus genes are organized into gene families and here we describe the duplications that have led to the expansion of the largest bracovirus gene family that encode PTPs.

Our data suggest that PTP gene family expansion occurred via four mechanisms: by duplication of large segments of the chromosomally integrated form of the virus sequences (segmental duplication), by tandem duplications within this form and by dispersed duplications. We also highlight a novel mechanism of duplication specific to PDVs that involves viral circle reintegration into the wasp genome. The PTP copies produced by duplications were shown to undergo conservative evolution along with episodes of adaptive evolution. In particular, recently produced copies have undergone positive selection in sites involved in defining substrate selectivity in vertebrate PTPs suggesting their implication in ongoing adaptation.

Taken together our results provide evidence of the dynamic nature of polydnavirus proviral genomes and reveal that these viruses could be sources of new genes and possibly new functions for insects via a specific circle reintegration mechanism.

## Methods

### Newly isolated PTPs

The homologues of 14 CcBV PTP genes previously identified in the CcBV genome corresponding to PTP P, Q, Y, K, L, C, α, S, M, E, X, H, R and Δ [29] have been newly isolated and sequenced from bracoviruses associated with other *Cotesia* species, in order to increase the data set for selection analyses. Eighty-two PTP genes (see gene accession numbers and abbreviations in Additional file 6) were isolated from eight *Cotesia* species: *C. chilonis* (laboratory reared, USA, Wiedenmann R), *C. flavipes* (field collected, Kenya, Dupas S), *C. glomerata* (laboratory reared, Netherlands, Vet L), *C. melanoscela* (field collected, France, Villemant C), *C. marginiventris* (laboratory reared, USA, Joyce A), *C. vestalis* (field collected, Benin, Guilloux T), *C. rubecula* (laboratory reared, Netherland, Smid H), *C. sesamiae* (field collected, Kenya, Dupas S). All specimens were preserved in 95% ethanol and maintained at –20°C until DNA was extracted.

### DNA extraction, amplification and sequencing

DNA was extracted from 2 individuals for each species except for larger wasps (*C. rubecula:* 1 individual used). Individuals were ground in a 5% chelex 100 resin (Biorad) solution with proteinase K (0.12 mg/ml) and incubated at 56°C for 30 min, then incubated at 95°C for 15 min and supernatants were collected. The primers were designed based upon the sequences of the CcBV PTPs: each pair of primers is specific for each gene and enables the amplification of a DNA sequence encoding the 10 conserved motifs that characterize PTPs. Primer sequences are listed in Additional file 7. PCR conditions using Goldstar (Eurogentec) varied depending on whether PTP genes were amplified from species closely related to *Cotesia congregata* (55°C annealing temperature and 1.5 mM of MgCl_2_) or from more distantly related species (annealing at 45°C and 3 mM of MgCl_2_). One microliter of DNA was used for each PCR reaction. The standard PCR program was composed of a first denaturation step (95°C for 2.5 min) followed by 30 cycles of amplification (denaturation at 95°C for 30 sec, annealing for 45 sec, elongation at 72°C for 60 sec) and a final elongation step (72°C for 5 min). The PCR products were purified with the Qiaquick kit (Qiagen) and sequenced directly. We always obtained a minimum of two identical sequences for those reported. For PTP C and α and PTP E and X, sequence profiles showed multiple peaks, indicating that mixed alleles were amplified due to the close relationship between gene copies, thus PCR products were cloned into the pGEMT vector (Qiagen cloning kit) and 10 clones were sequenced. The sequencing reactions were performed with the BigDye Terminator Sequencing Kit (Perkin Elmer ABI) and analysed on an ABI PRISM 3100 Genetic Analyzer.

### Sequence analysis and phylogeny of PTPs (newly isolated or retrieved from Genbank)

We also searched for homologous PTP genes in CcBV, CvBV (*Cotesia vestalis* bracovirus previously known as *Cotesia plutellae* bracovirus), GiBV, GfBV and MdBV in public databanks (see accession numbers in Additional file 6). Twenty-eight PTP sequences from CvBV, 42 sequences from GiBV, 32 sequences from GfBV, 13 sequences from MdBV and 27 sequences from CcBV were retrieved at NCBI (http://blast.ncbi.nlm.nih.gov/Blast.cgi). Both retrieved and newly isolated PTPs were used for phylogenetic and selection analyses. For the analysis of duplications, the maps of the PTP genes on the virus segments (Figure 2, Additional files 2, 3 and 4) are represented based on gene positions indicated in Genbank.

ClustalX alignment [67] of translated sequences were corrected manually based on the PTP conserved motifs [28]. This alignment (Additional file 5) was then submitted to Gblocks0.91b [68] in order to eliminate poorly aligned positions and divergent regions that could be misleading in phylogenetic analyses.

This sequence alignment was used to construct a tree in order to give an overview of PTP evolution in Microgastrinae. The GTR + I + G model of sequence evolution was selected for most clades using Modeltest version 3.7 [69] according to the likelihood ratio test (LRT) and the Akaike information criterion (AIC). For the IZCα clade the HKY 85 + G model was selected. Bayesian MCMC analyses were performed for the entire data set using MrBayes version 3.12 [70]. Two independent analyses were run simultaneously for each data set, each consisting of 10^6^ generations, sampled every 10^3^ generations and using four chains and uniform priors. Maximum likelihood analysis (ML) was performed on PHYML program [71] using the same evolutionary model. The topology and the branch length estimations were repeated 1000 times for bootstrap test.

### Branch and site selection analyses

Consensus trees were chosen as a phylogenetic hypothesis for the estimation of nonsynonymous to synonymous substitution rate ratio (ω = dN/dS) models on each clade using PAML 4.2 [72]. Six different models of site- and/or branch-specific ω ratios [73,74] were optimised using Bayesian methods in PAML 4.2 [72]. The maximum likelihoods were compared between nested models by the Likelihood Ratio Test (LRT).

Site-specific positive selection was tested by comparing the selective model M8 (ω>1) to the non-selective model M8a (ω = 1) by LRT [75]. Branch specific selection was tested by comparing models M0b (branch specific selection and no variation among sites) to M0 (no branch or site-specific selection) using an LRT. Finally, the branch + site models were developed to address positive selection at a subset of sites on branches specified *a priori*. Model MA defines four classes of sites, where the two last classes have ω > 1 on the lineage of interest and ω < 1 for the rest of branches. This model is compared with MAnull which imposes ω = 1 for the latter two classes.

## Abbreviations

PDVs: Polydnavirus; PTPs: Protein Tyrosine Phosphatases; BVs: Bracoviruses; IVs: Ichnoviruses; CcBV: *Cotesia congregata* Bracovirus; Mya: million years ago; CiBV: *Chelonus inanitus* Bracovirus; GfV: *Glypta fumiferana* Virus; CvBV: *Cotesia vestalis* Bracovirus; MdBV: *Microplitis demolitor* Bracovirus; GiBV: *Glyptapanteles indiensis* Bracovirus; GfBV: *Glyptapanteles flavicoxis* Bracovirus; UTR: UnTranslated Region; LRT: Likelihood Ratio Test; DRJ: Direct Repeat Jonction; AIC: Akaike Information Criterion; MCMC: Markov Chain Monte Carlo; PAML: Phylogenetic Analysis by Maximum Likelihood.

## Competing interests

The authors declare no competing interests.

## Authors’ contributions

CS performed phylogenetic and PAML analyses and wrote the first draft of the publication. SD initiated the PAML analysis. EP performed phylogenetic and PAML analyses and completed the final draft. FH obtained the new sequence data. CD analysed the reintegrated form in *C. sesamiae*. EH and J-MD supervised the work and were involved in writing the publication. All authors read and approved the final manuscript.

## Supplementary Material

Additional file 1 Unrooted PTP phylogenetic tree from Bayesian inferences under the GTR + I + G substitution model and Maximum Likelihood with all sequence names (please use the enlargment tool of your browser to visualize the smallest typing). Click here for file

Additional file 2 **Summary of PTP genes organization in different bracovirus genomes.** Segments known to be isolated in the wasp genome are indicated by a star (CvBV organization in the wasp genome is unknown). Click here for file

Additional file 3 Orthologous genomic regions from CcBV, CvBV, GiBV and GfBV(A) Orthologous genes of CcBV circle 14, GiBV segment 26 and GfBV segment 26, (B) Orthologous genes of CcBV circle 4, CvBV segment S50, GiBV segment 28 and GfBV segment 27 and 28. Click here for file

Additional file 4 Paralogous relationships between: CcBV circle 1 and CcBV circle 17 and 10 (A), *Glyptapanteles indiensis *segment 24 and 30 (B). Click here for file

Additional file 5 **Alignment of the 239 PTP sequences used for analyses (input file used for Gblock).** Stars indicate stop codons. Click here for file

Additional file 6 PTP accession numbers. Click here for file

Additional file 7 **Primers used for PTP amplification in different *Cotesia species.*** Click here for file
