# Supplementary figures and images for: Evolutionary mechanisms driving the evolution of a large polydnavirus gene family coding for protein tyrosine phosphatases
Source: BMC Evol Biol. 2012 Dec 27;12:253. doi: 10.1186/1471-2148-12-253 (PMC3573978; doi:10.1186/1471-2148-12-253)

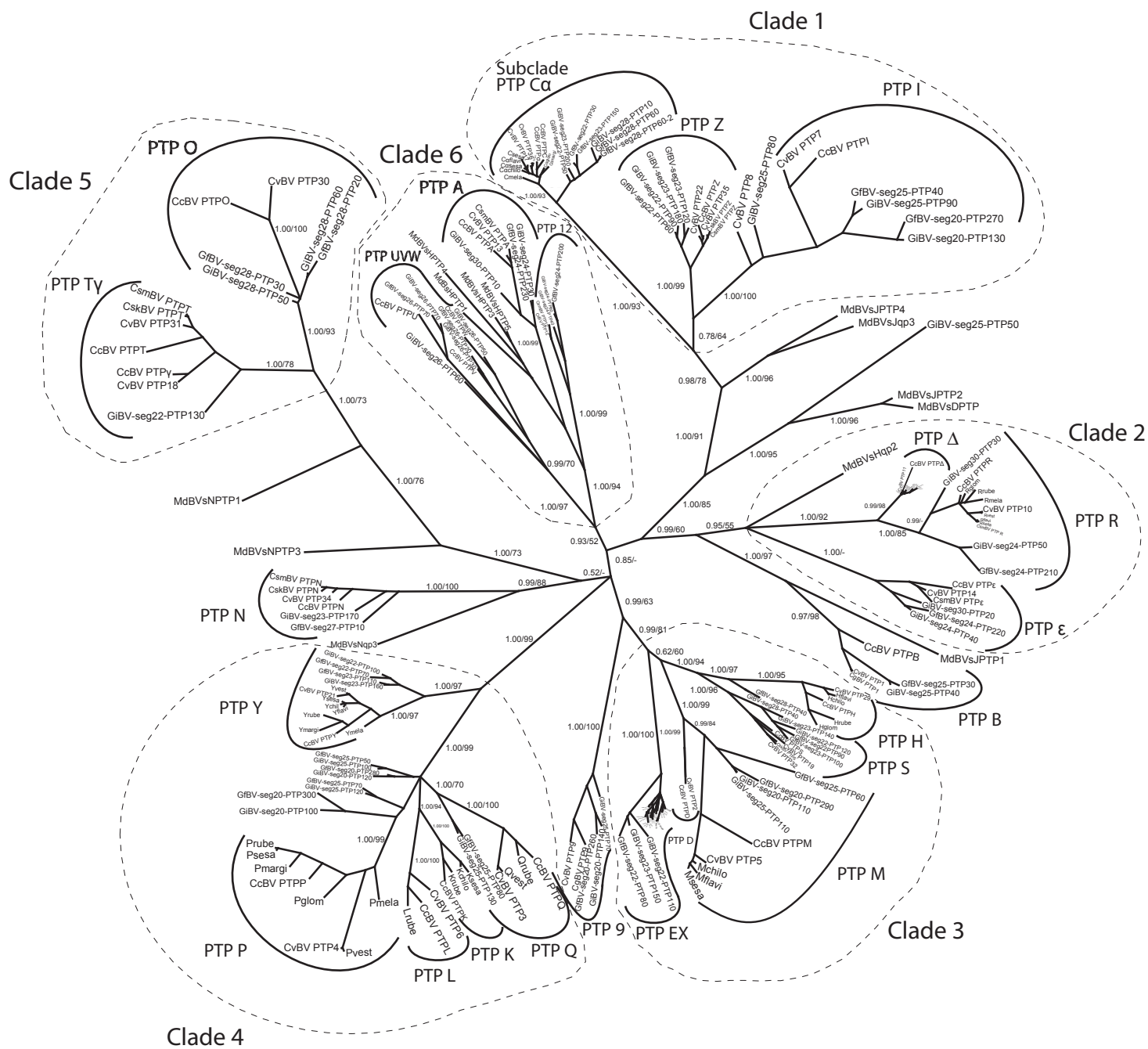

0.1 substitutions/site

Supplement: Additional file 1 — Unrooted PTP phylogenetic tree from Bayesian inferences under the GTR + I + G substitution model and Maximum Likelihood with all sequence names (please use the enlargment tool of your browser to visualize the smallest typing). [file 1471-2148-12-253-S1.pdf]

A) CcBV circles

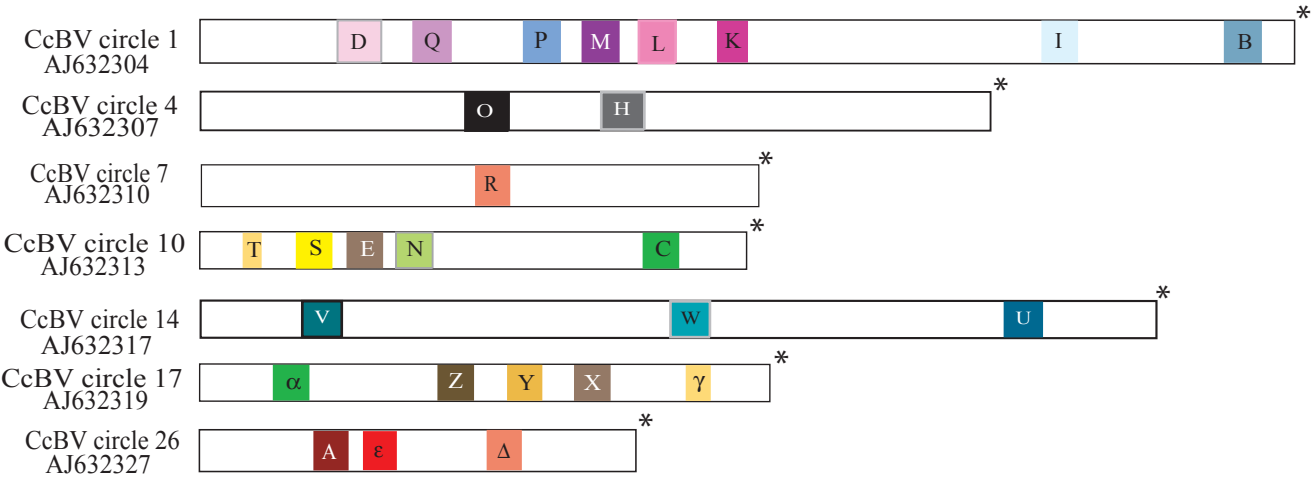

B) CvBV segments

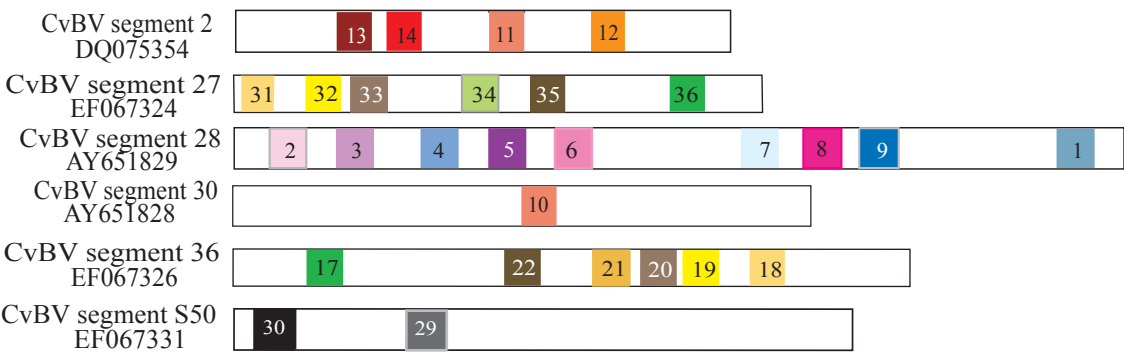

C) GiBV segments

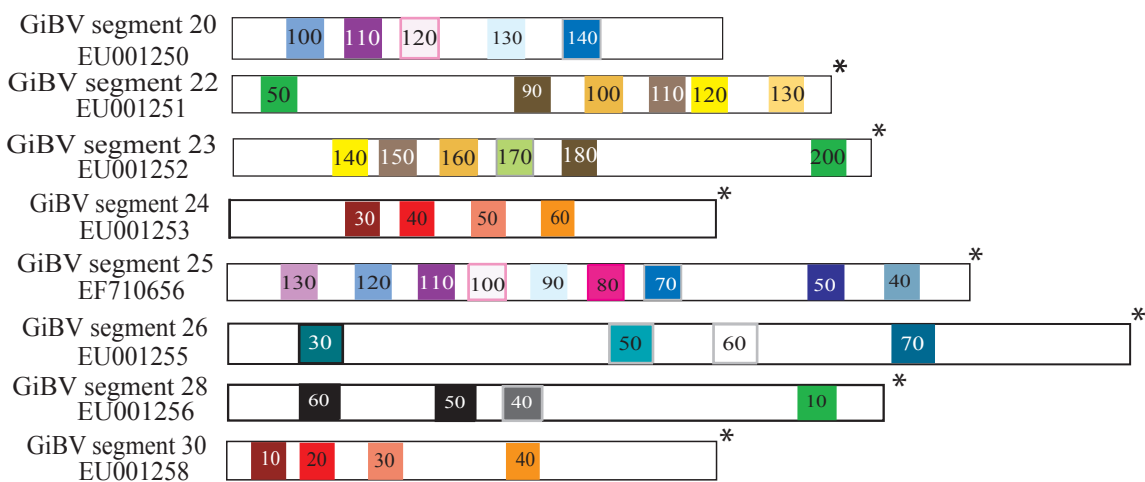

D) GfBV segments

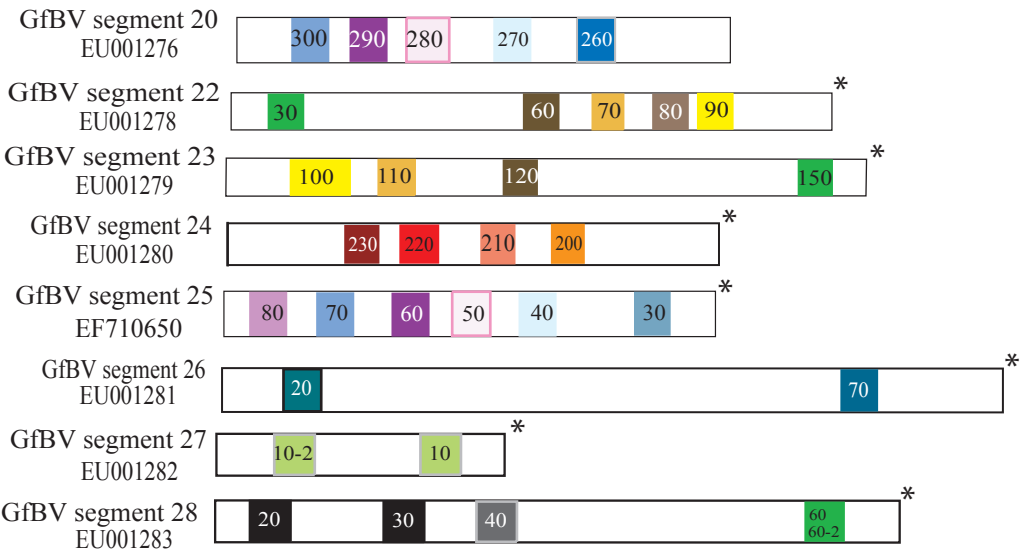

Supplement: Additional file 2 — Summary of PTP genes organization in different bracovirus genomes. Segments known to be isolated in the wasp genome are indicated by a star (CvBV organization in the wasp genome is unknown). [file 1471-2148-12-253-S2.pdf]

A)

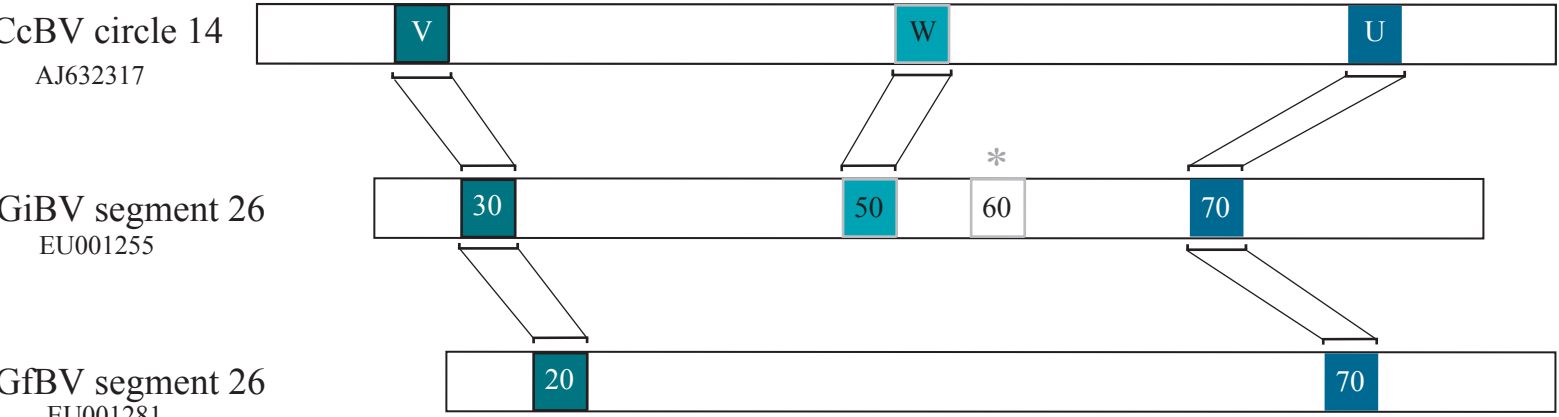

B)

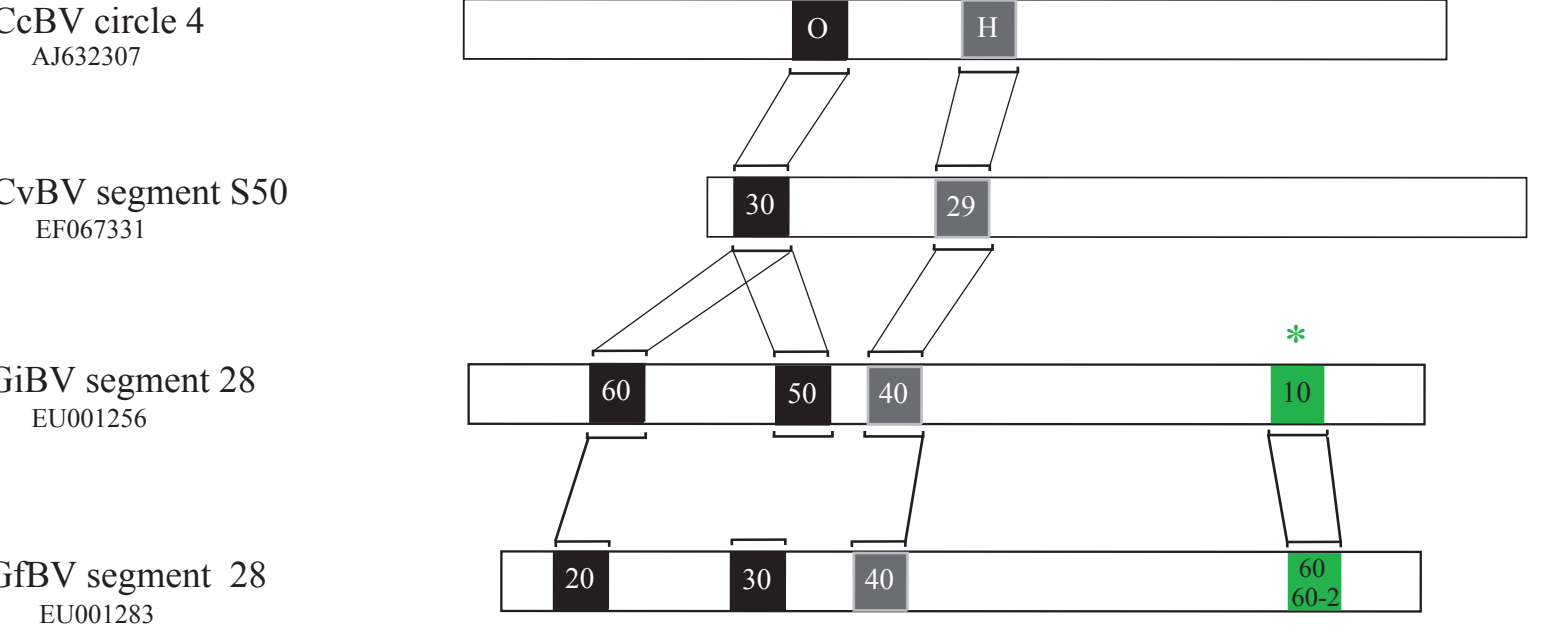

Supplement: Additional file 3 — Orthologous genomic regions from CcBV, CvBV, GiBV and GfBV(A) Orthologous genes of CcBV circle 14, GiBV segment 26 and GfBV segment 26, (B) Orthologous genes of CcBV circle 4, CvBV segment S50, GiBV segment 28 and GfBV segment 27 and 28. [file 1471-2148-12-253-S3.pdf]

A)

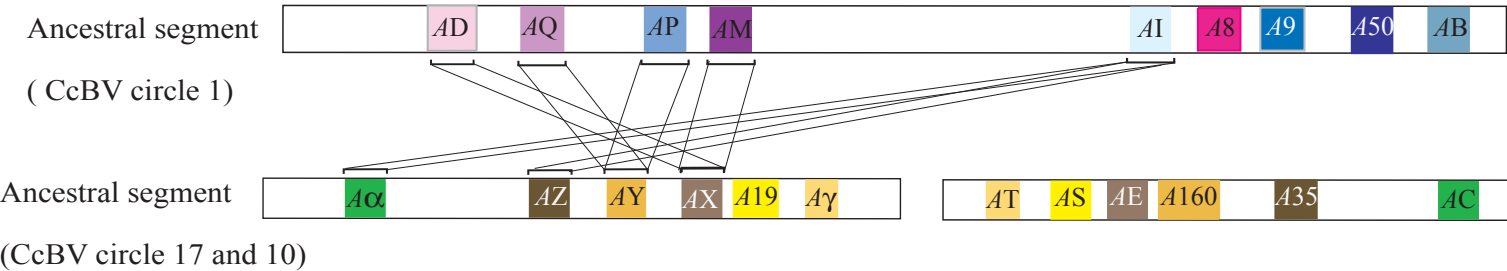

B)

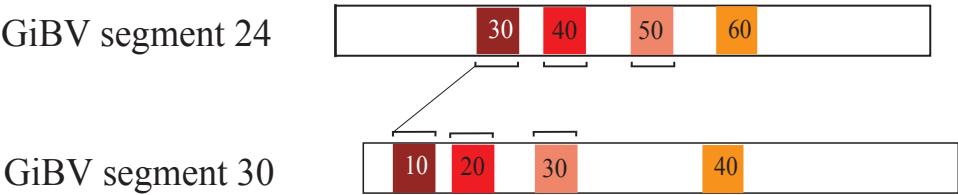

Supplement: Additional file 4 — Paralogous relationships between: CcBV circle 1 and CcBV circle 17 and 10 (A), Glyptapanteles indiensis segment 24 and 30 (B). [file 1471-2148-12-253-S4.pdf]
