# Supplementary material for: Evolutionary mechanisms driving the evolution of a large polydnavirus gene family coding for protein tyrosine phosphatases
Source: BMC Evol Biol. 2012 Dec 27;12:253. doi: 10.1186/1471-2148-12-253 (PMC3573978; doi:10.1186/1471-2148-12-253)
Supplement: Additional file 5 — Alignment of the 239 PTP sequences used for analyses (input file used for Gblock). Stars indicate stop codons. [file 1471-2148-12-253-S5.pdf]

Alignment: F:\Alignementfinal\final9modprot2nex.txt
Seaview [blocks=10 fontsize=6 A4] on Mon Jul 25 14:48:06 2011

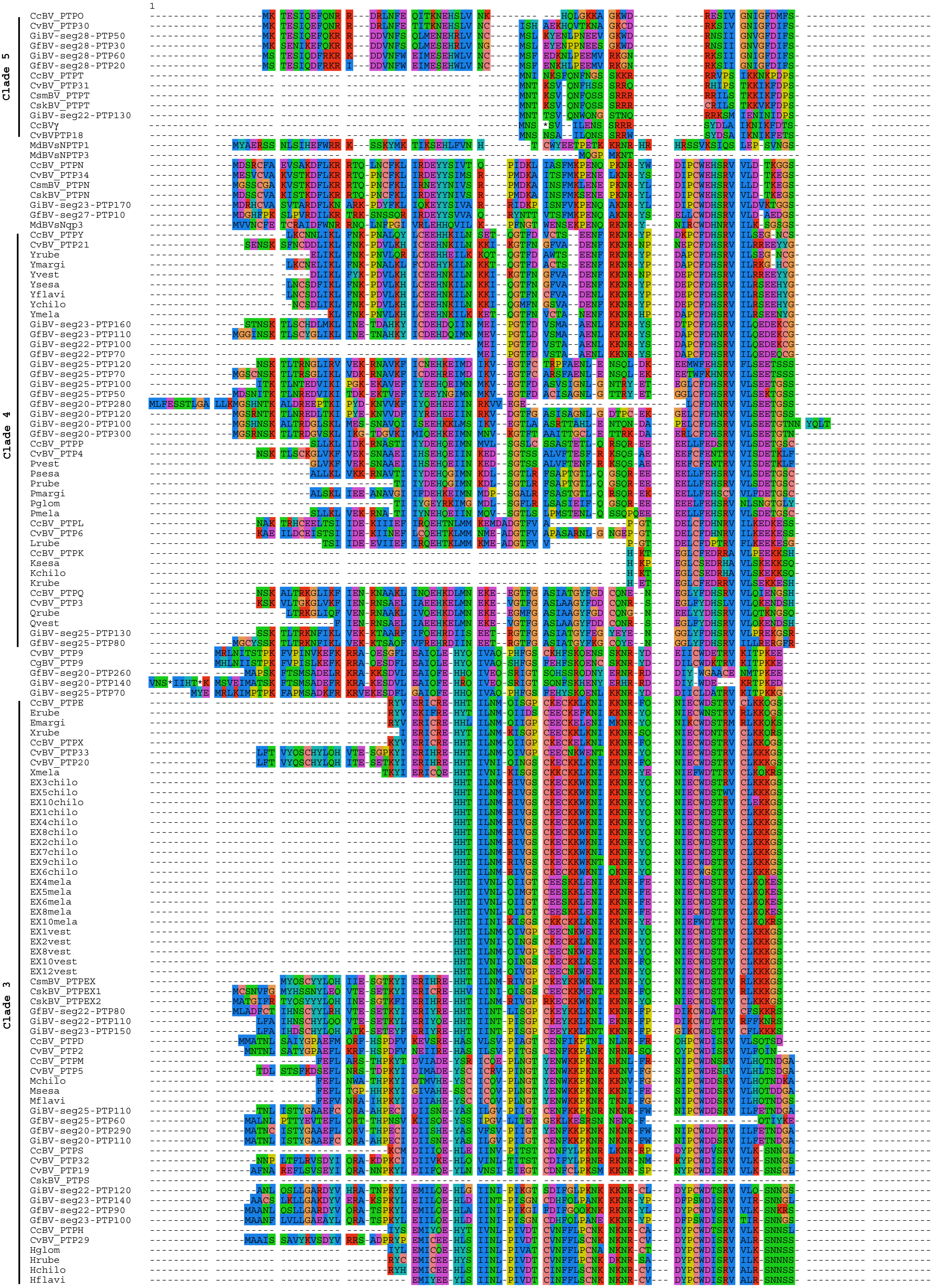

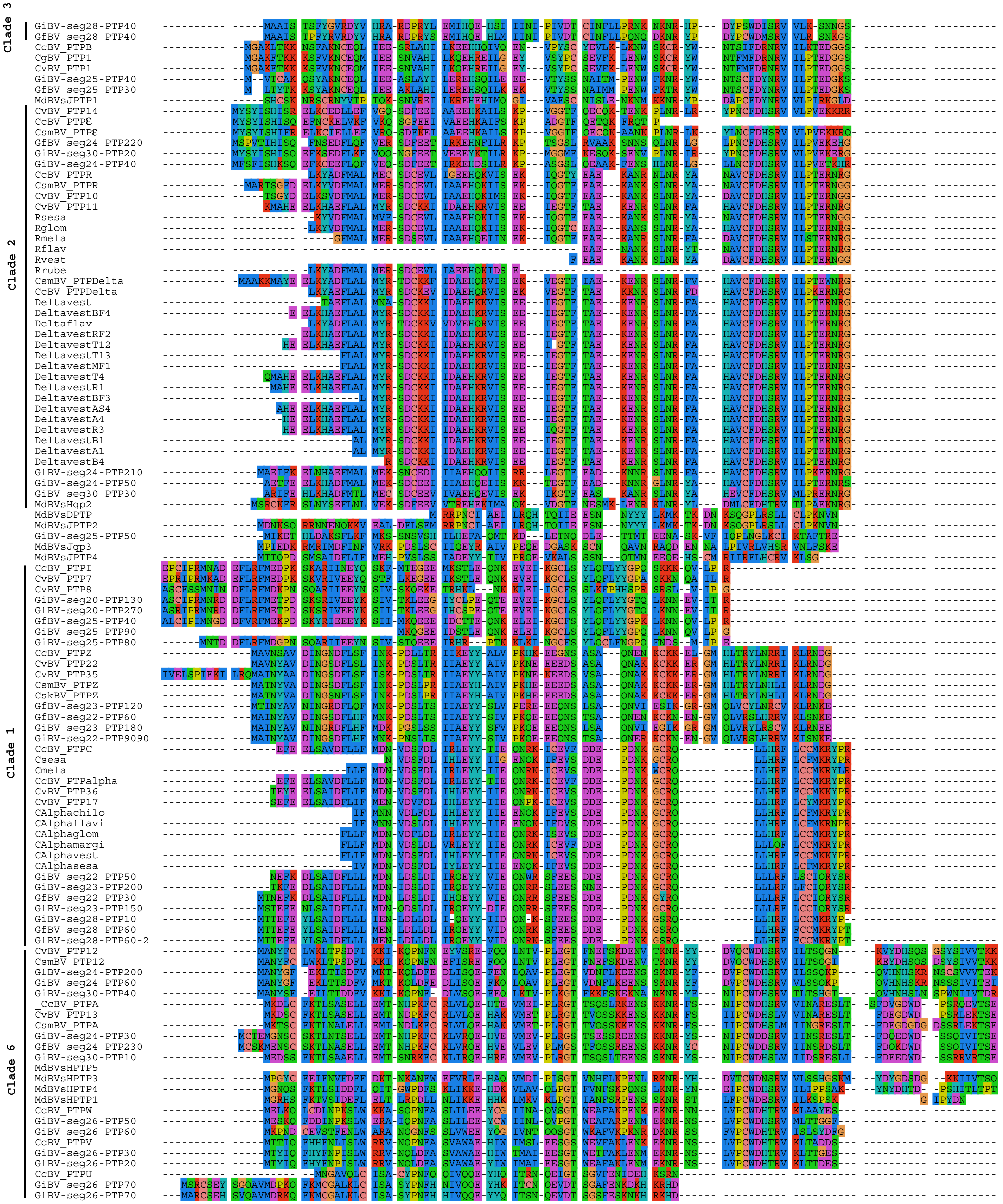

Clade 3
GiBV-seg28-PTP40
GfBV-seg28-PTP40
CcBV_PTPB
CgBV_PTP1
CvBV_PTP1
GiBV-seg25-PTP40
GfBV-seg25-PTP30
MdBVsJPTP1
Clade 2
CvBV_PTP14
CcBV_PTPƐ
CsmBV_PTPƐ
GfBV-seg24-PTP220
GiBV-seg30-PTP20
GiBV-seg24-PTP40
CcBV_PTPR
CsmBV_PTPR
CvBV_PTP10
CvBV_PTP11
Rsesa
Rglom
Rmela
Rflav
Rvest
Rrube
CsmBV_PTPDelta
CcBV_PTPDelta
Deltavest
DeltavestBF4
Deltaflav
DeltavestRF2
DeltavestT12
DeltavestT13
DeltavestMF1
DeltavestT4
DeltavestR1
DeltavestBF3
DeltavestAS4
DeltavestA4
DeltavestR3
DeltavestB1
DeltavestA1
DeltavestB4
GfBV-seg24-PTP210
GiBV-seg24-PTP50
GiBV-seg30-PTP30
MdBVsHqp2
MdBVsDPTP
MdBVsJPTP2
GiBV-seg25-PTP50
MdBVsJqp3
MdBVsJPTP4
Clade 1
CcBV_PTPI
CvBV_PTP7
CvBV_PTP8
GiBV-seg20-PTP130
GfBV-seg20-PTP270
GfBV-seg25-PTP40
GiBV-seg25-PTP90
GiBV-seg25-PTP80
CcBV_PTPZ
CvBV_PTP22
CvBV_PTP35
CsmBv_PTPZ
CskBV_PTPZ
GfBV-seg23-PTP120
GfBV-seg22-PTP60
GiBV-seg23-PTP180
GiBV-seg22-PTP9090
CcBV_PTPC
Csesa
Cmela
CcBV_PTPalpha
CvBV_PTP36
CvBV_PTP17
CAlphachilo
CAlphaflavi
CAlphaglom
CAlphamargi
CAlphavest
CAlphasesa
GiBV-seg22-PTP50
GiBV-seg23-PTP200
GfBV-seg22-PTP30
GfBV-seg23-PTP150
GiBV-seg28-PTP10
GfBV-seg28-PTP60
GfBV-seg28-PTP60-2
Clade 6
CvBV_PTP12
CsmBV_PTP12
GfBV-seg24-PTP200
GiBV-seg24-PTP60
GiBV-seg30-PTP40
_CcBV_PTPA
CvBV_PTP13
CsmBV_PTPA
GiBV-seg24-PTP30
GfBV-seg24-PTP230
GiBV-seg30-PTP10
MdBVsHPTP5
MdBVsHPTP3
MdBVsHPTP4
MdBVsHPTP1
CcBV_PTPW
GiBV-seg26-PTP50
GiBV-seg26-PTP60
CcBV_PTPV
GiBV-seg26-PTP30
GfBV-seg26-PTP20
CcBV_PTPU
GiBV-seg26-PTP70
GfBV-seg26-PTP70

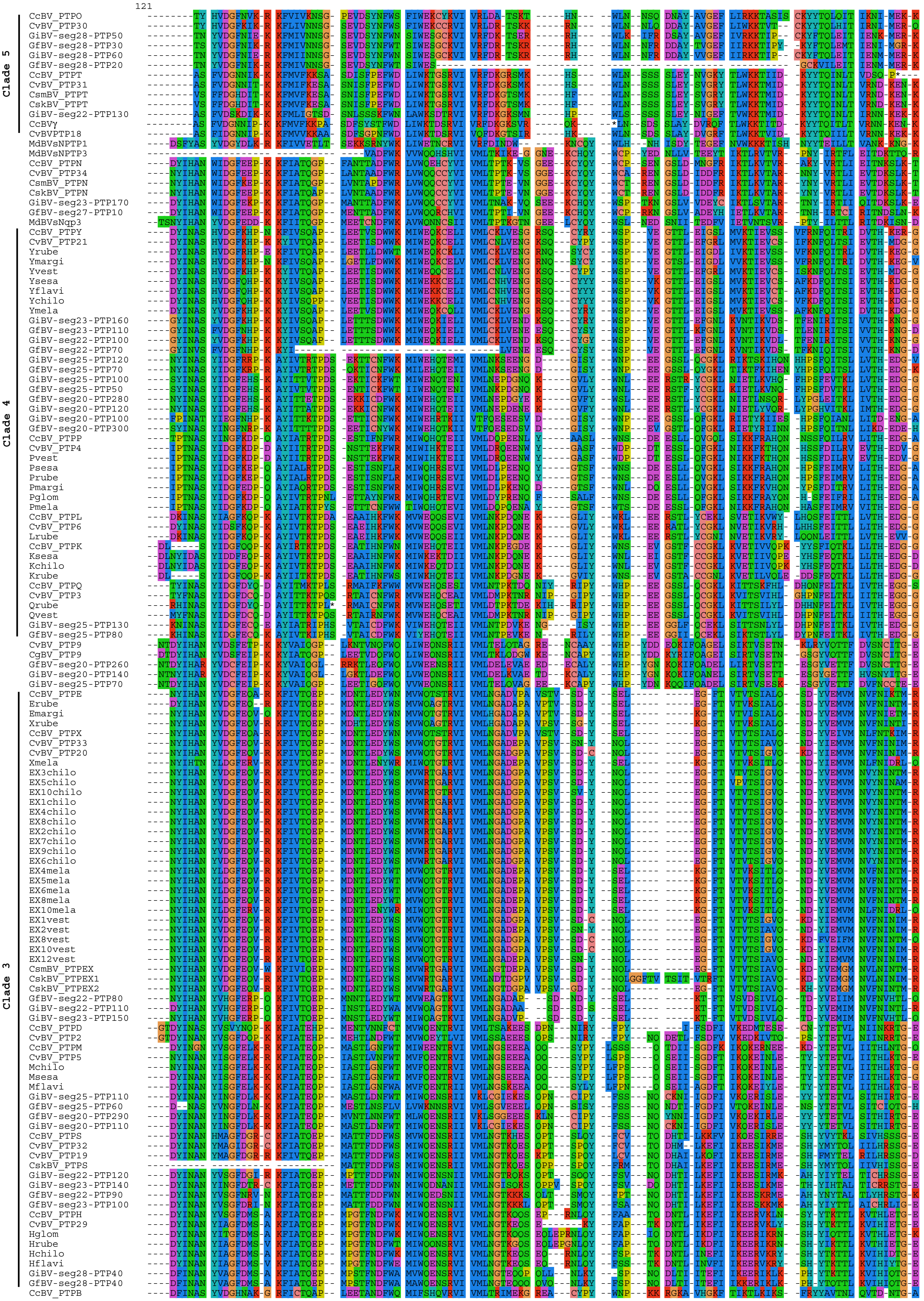
121
Clade 5
Clade 4
Clade 3
CcBV_PTPO
CvBV_PTP30
GiBV-seg28-PTP50
GfBV-seg28-PTP30
GiBV-seg28-PTP60
GfBV-seg28-PTP20
CcBV_PTPT
CvBV_PTP31
CsmBV_PTPT
CskBV_PTPT
GiBV-seg22-PTP130
CcBVγ
CvBVPTP18
MdBVsNPTP1
MdBVsNPTP3
CcBV_PTPN
CvBV_PTP34
CsmBV_PTPN
CskBV_PTPN
GiBV-seg23-PTP170
GfBV-seg27-PTP10
MdBVsNqp3
CcBV_PTPY
CvBV_PTP21
Yrube
Ymargi
Yvest
Ysesa
Yflavi
Ychilo
Ymela
GiBV-seg23-PTP160
GfBV-seg23-PTP110
GiBV-seg22-PTP100
GfBV-seg22-PTP70
GiBV-seg25-PTP120
GfBV-seg25-PTP70
GiBV-seg25-PTP100
GfBV-seg25-PTP50
GfBV-seg20-PTP280
GiBV-seg20-PTP120
GiBV-seg20-PTP100
GfBV-seg20-PTP300
CcBV_PTPP
CvBV_PTP4
Pvest
Psesa
Prube
Pmargi
Pglom
Pmela
CcBV_PTPL
CvBV_PTP6
Lrube
CcBV_PTPK
Ksesa
Kchilo
Krube
CcBV_PTPQ
CvBV_PTP3
Qrube
Qvest
GiBV-seg25-PTP130
GfBV-seg25-PTP80
CvBV_PTP9
CgBV_PTP9
GfBV-seg20-PTP260
GiBV-seg20-PTP140
GiBV-seg25-PTP70
CcBV_PTPE
Erube
Emargi
Xrube
CcBV_PTPX
CvBV_PTP33
CvBV_PTP20
Xmela
EX3chilo
EX5chilo
EX10chilo
EX1chilo
EX4chilo
EX8chilo
EX2chilo
EX7chilo
EX9chilo
EX6chilo
EX4mela
EX5mela
EX6mela
EX8mela
EX10mela
EX1vest
EX2vest
EX8vest
EX10vest
EX12vest
CsmBV_PTPEX
CskBV_PTPEX1
CskBV_PTPEX2
GfBV-seg22-PTP80
GiBV-seg22-PTP110
GiBV-seg23-PTP150
CcBV_PTPD
CvBV_PTP2
CcBV_PTPM
CvBV_PTP5
Mchilo
Msesa
Mflavi
GiBV-seg25-PTP110
GfBV-seg25-PTP60
GfBV-seg20-PTP290
GiBV-seg20-PTP110
CcBV_PTPS
CvBV_PTP32
CvBV_PTP19
CskBV_PTPS
GiBV-seg22-PTP120
GiBV-seg23-PTP140
GfBV-seg22-PTP90
GfBV-seg23-PTP100
CcBV_PTPH
CvBV_PTP29
Hglom
Hrube
Hchilo
Hflavi
GiBV-seg28-PTP40
GfBV-seg28-PTP40
CcBV_PTPB

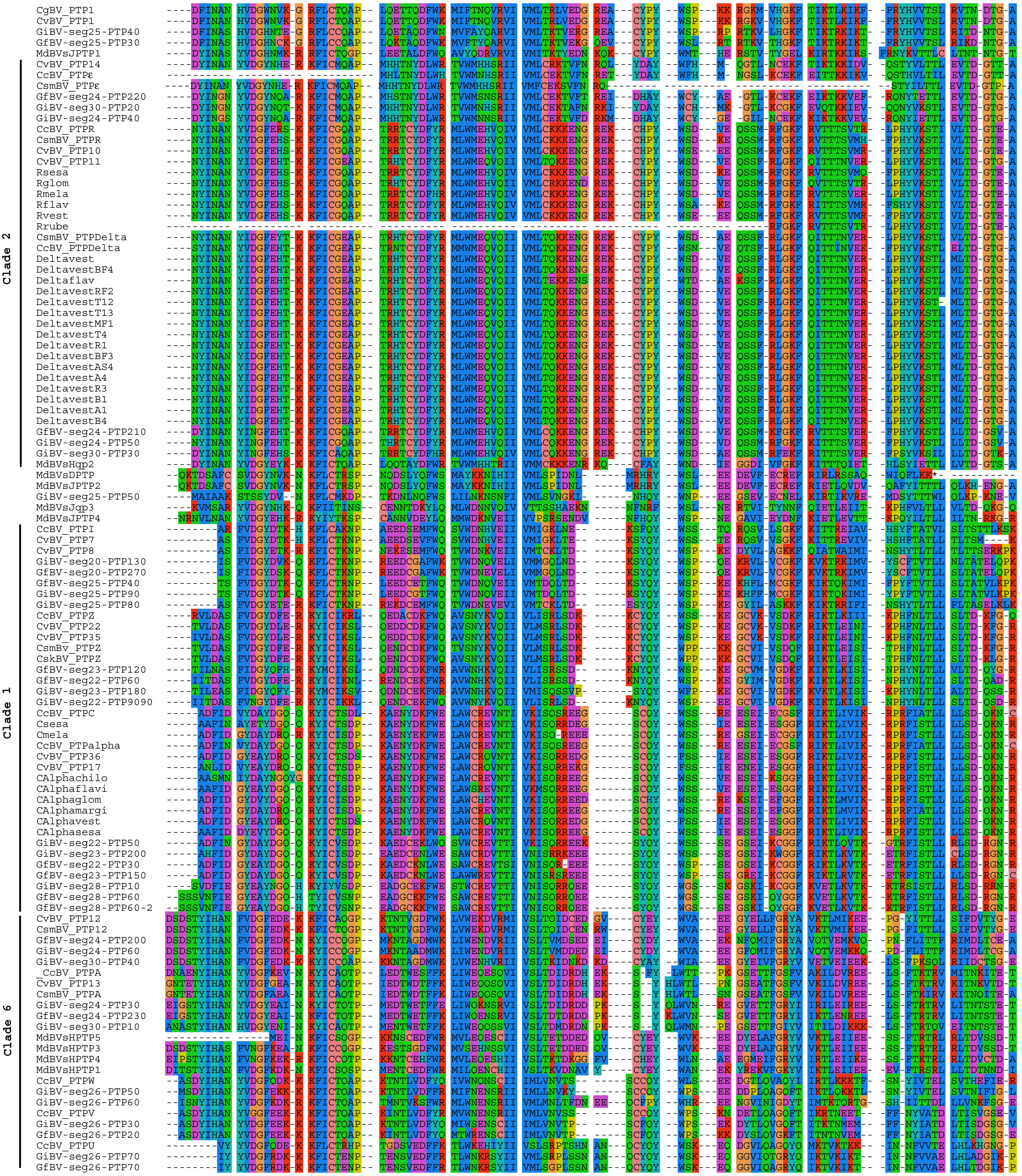

Clade 2
Clade 1
Clade 6
CgBV_PTP1
CvBV_PTP1
GiBV-seg25-PTP40
GfBV-seg25-PTP30
MdBVsJPTP1
CvBV_PTP14
CcBV_PTPε
CsmBV_PTPε
GfBV-seg24-PTP220
GiBV-seg30-PTP20
GiBV-seg24-PTP40
CcBV_PTPR
CsmBV_PTPR
CvBV_PTP10
CvBV_PTP11
Rsesa
Rglom
Rmela
Rflav
Rvest
Rrube
CsmBV_PTPDelta
CcBV_PTPDelta
Deltavest
DeltavestBF4
Deltaflav
DeltavestRF2
DeltavestT12
DeltavestT13
DeltavestMF1
DeltavestT4
DeltavestR1
DeltavestBF3
DeltavestAS4
DeltavestA4
DeltavestR3
DeltavestB1
DeltavestA1
DeltavestB4
GfBV-seg24-PTP210
GiBV-seg24-PTP50
GiBV-seg30-PTP30
MdBVsHqp2
MdBVsDPTP
MdBVsJPTP2
GiBV-seg25-PTP50
MdBVsJqp3
MdBVsJPTP4
CcBV_PTPI
CvBV_PTP7
CvBV_PTP8
GiBV-seg20-PTP130
GfBV-seg20-PTP270
GfBV-seg25-PTP40
GiBV-seg25-PTP90
GiBV-seg25-PTP80
CcBV_PTPZ
CvBV_PTP22
CvBV_PTP35
CsmBv_PTPZ
CskBV_PTPZ
GfBV-seg23-PTP120
GfBV-seg22-PTP60
GiBV-seg23-PTP180
GiBV-seg22-PTP9090
CcBV_PTPC
Csesa
Cmela
CcBV_PTPalpha
CvBV_PTP36
CvBV_PTP17
CAlphachilo
CAlphaflavi
CAlphaglom
CAlphamargi
CAlphavest
CAlphasesa
GiBV-seg22-PTP50
GiBV-seg23-PTP200
GfBV-seg22-PTP30
GfBV-seg23-PTP150
GiBV-seg28-PTP10
GfBV-seg28-PTP60
GfBV-seg28-PTP60-2
CvBV_PTP12
CsmBV_PTP12
GfBV-seg24-PTP200
GiBV-seg24-PTP60
GiBV-seg30-PTP40
_CcBV_PTPA
CvBV_PTP13
CsmBV_PTPA
GiBV-seg24-PTP30
GfBV-seg24-PTP230
GiBV-seg30-PTP10
MdBVsHPTP5
MdBVsHPTP3
MdBVsHPTP4
MdBVsHPTP1
CcBV_PTPW
GiBV-seg26-PTP50
GiBV-seg26-PTP60
CcBV_PTPV
GiBV-seg26-PTP30
GfBV-seg26-PTP20
CcBV_PTPU
GiBV-seg26-PTP70
GfBV-seg26-PTP70

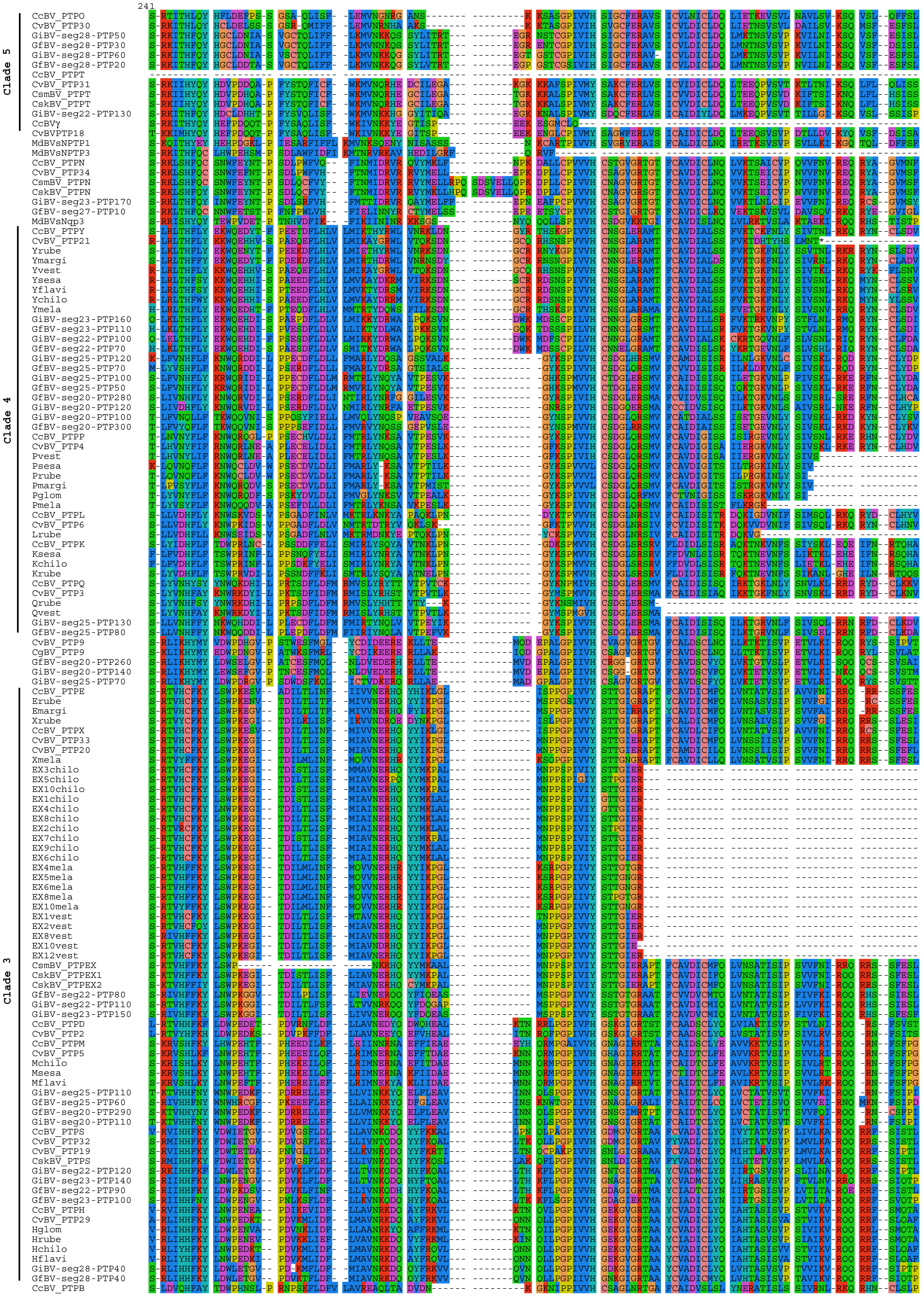
241
Clade 5
Clade 4
Clade 3
CcBV_PTPO
CvBV_PTP30
GiBV-seg28-PTP50
GfBV-seg28-PTP30
GiBV-seg28-PTP60
GfBV-seg28-PTP20
CcBV_PTPT
CvBV_PTP31
CsmBV_PTPT
CskBV_PTPT
GiBV-seg22-PTP130
CcBVγ
CvBVPTP18
MdBVsNPTP1
MdBVsNPTP3
CcBV_PTPN
CvBV_PTP34
CsmBV_PTPN
CskBV_PTPN
GiBV-seg23-PTP170
GfBV-seg27-PTP10
MdBVsNqp3
CcBV_PTPY
CvBV_PTP21
Yrube
Ymargi
Yvest
Ysesa
Yflavi
Ychilo
Ymela
GiBV-seg23-PTP160
GfBV-seg23-PTP110
GiBV-seg22-PTP100
GfBV-seg22-PTP70
GiBV-seg25-PTP120
GfBV-seg25-PTP70
GiBV-seg25-PTP100
GfBV-seg25-PTP50
GfBV-seg20-PTP280
GiBV-seg20-PTP120
GiBV-seg20-PTP100
GfBV-seg20-PTP300
CcBV_PTPP
CvBV_PTP4
Pvest
Psesa
Prube
Pmargi
Pglom
Pmela
CcBV_PTPL
CvBV_PTP6
Lrube
CcBV_PTPK
Ksesa
Kchilo
Krube
CcBV_PTPQ
CvBV_PTP3
Qrube
Qvest
GiBV-seg25-PTP130
GfBV-seg25-PTP80
CvBV_PTP9
CgBV_PTP9
GfBV-seg20-PTP260
GiBV-seg20-PTP140
GiBV-seg25-PTP70
CcBV_PTPE
Erube
Emargi
Xrube
CcBV_PTPX
CvBV_PTP33
CvBV_PTP20
Xmela
EX3chilo
EX5chilo
EX10chilo
EX1chilo
EX4chilo
EX8chilo
EX2chilo
EX7chilo
EX9chilo
EX6chilo
EX4mela
EX5mela
EX6mela
EX8mela
EX10mela
EX1vest
EX2vest
EX8vest
EX10vest
EX12vest
CsmBV_PTPEX
CskBV_PTPEX1
CskBV_PTPEX2
GfBV-seg22-PTP80
GiBV-seg22-PTP110
GiBV-seg23-PTP150
CcBV_PTPD
CvBV_PTP2
CcBV_PTPM
CvBV_PTP5
Mchilo
Msesa
Mflavi
GiBV-seg25-PTP110
GfBV-seg25-PTP60
GfBV-seg20-PTP290
GiBV-seg20-PTP110
CcBV_PTPS
CvBV_PTP32
CvBV_PTP19
CskBV_PTPS
GiBV-seg22-PTP120
GiBV-seg23-PTP140
GfBV-seg22-PTP90
GfBV-seg23-PTP100
CcBV_PTPH
CvBV_PTP29
Hglom
Hrube
Hchilo
Hflavi
GiBV-seg28-PTP40
GfBV-seg28-PTP40
CcBV_PTPB

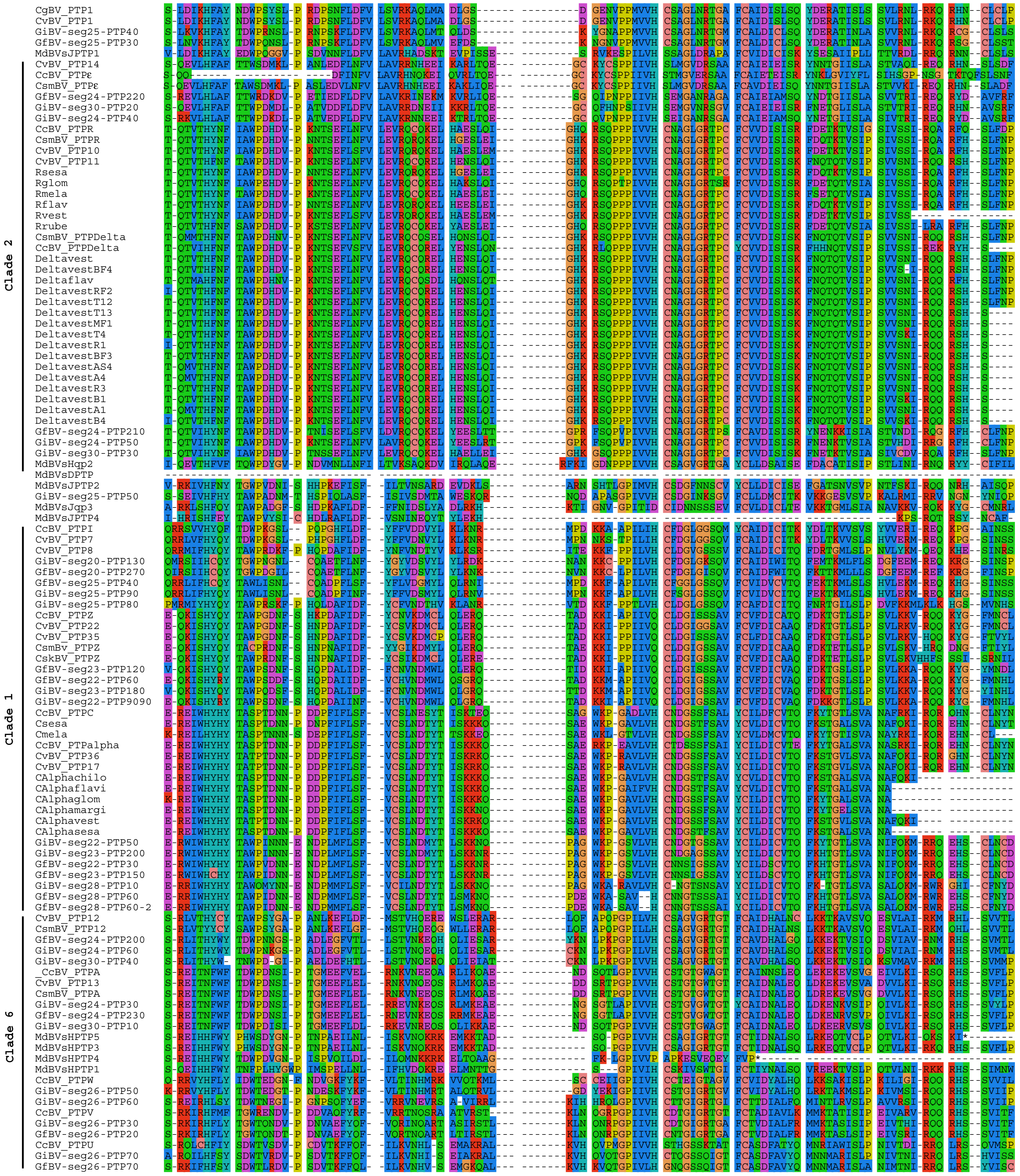

CgBV_PTP1
CvBV_PTP1
GiBV-seg25-PTP40
GfBV-seg25-PTP30
MdBVsJPTP1
CvBV_PTP14
CcBV_PTPε
CsmBV_PTPε
GfBV-seg24-PTP220
GiBV-seg30-PTP20
GiBV-seg24-PTP40
CcBV_PTPR
CsmBV_PTPR
CvBV_PTP10
CvBV_PTP11
Rsesa
Rglom
Rmela
Rflav
Rvest
Rrube
CsmBV_PTPDelta
CcBV_PTPDelta
Deltavest
DeltavestBF4
Deltaflav
DeltavestRF2
DeltavestT12
DeltavestT13
DeltavestMF1
DeltavestT4
DeltavestR1
DeltavestBF3
DeltavestAS4
DeltavestA4
DeltavestR3
DeltavestB1
DeltavestA1
DeltavestB4
GfBV-seg24-PTP210
GiBV-seg24-PTP50
GiBV-seg30-PTP30
MdBVsHqp2
MdBVsDPTP
MdBVsJPTP2
GiBV-seg25-PTP50
MdBVsJqp3
MdBVsJPTP4
CcBV_PTPI
CvBV_PTP7
CvBV_PTP8
GiBV-seg20-PTP130
GfBV-seg20-PTP270
GfBV-seg25-PTP40
GiBV-seg25-PTP90
GiBV-seg25-PTP80
CcBV_PTPZ
CvBV_PTP22
CvBV_PTP35
CsmBv_PTPZ
CskBV_PTPZ
GfBV-seg23-PTP120
GfBV-seg22-PTP60
GiBV-seg23-PTP180
GiBV-seg22-PTP9090
CcBV_PTPC
Csesa
Cmela
CcBV_PTPalpha
CvBV_PTP36
CvBV_PTP17
CAlphachilo
CAlphaflavi
CAlphaglom
CAlphamargi
CAlphavest
CAlphasesa
GiBV-seg22-PTP50
GiBV-seg23-PTP200
GfBV-seg22-PTP30
GfBV-seg23-PTP150
GiBV-seg28-PTP10
GfBV-seg28-PTP60
GfBV-seg28-PTP60-2
CvBV_PTP12
CsmBV_PTP12
GfBV-seg24-PTP200
GiBV-seg24-PTP60
GiBV-seg30-PTP40
_CcBV_PTPA
CvBV_PTP13
CsmBV_PTPA
GiBV-seg24-PTP30
GfBV-seg24-PTP230
GiBV-seg30-PTP10
MdBVsHPTP5
MdBVsHPTP3
MdBVsHPTP4
MdBVsHPTP1
CcBV_PTPW
GiBV-seg26-PTP50
GiBV-seg26-PTP60
CcBV_PTPV
GiBV-seg26-PTP30
GfBV-seg26-PTP20
CcBV_PTPU
GiBV-seg26-PTP70
GfBV-seg26-PTP70
Clade 2
Clade 1
Clade 6

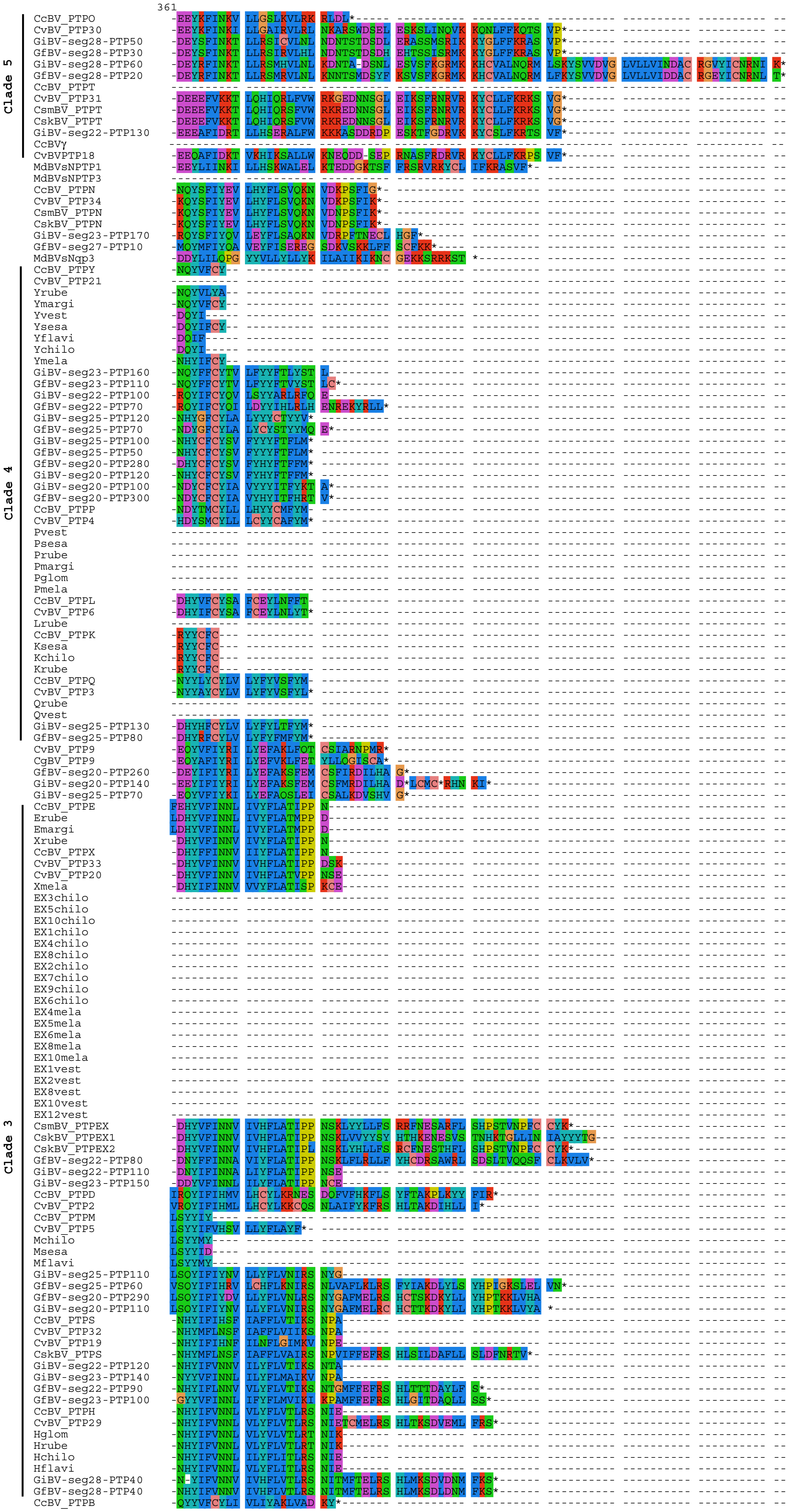

361
Clade 5
Clade 4
Clade 3
CcBV_PTPO
CvBV_PTP30
GiBV-seg28-PTP50
GfBV-seg28-PTP30
GiBV-seg28-PTP60
GfBV-seg28-PTP20
CcBV_PTPT
CvBV_PTP31
CsmBV_PTPT
CskBV_PTPT
GiBV-seg22-PTP130
CcBVγ
CvBVPTP18
MdBVsNPTP1
MdBVsNPTP3
CcBV_PTPN
CvBV_PTP34
CsmBV_PTPN
CskBV_PTPN
GiBV-seg23-PTP170
GfBV-seg27-PTP10
MdBVsNqp3
CcBV_PTPY
CvBV_PTP21
Yrube
Ymargi
Yvest
Ysesa
Yflavi
Ychilo
Ymela
GiBV-seg23-PTP160
GfBV-seg23-PTP110
GiBV-seg22-PTP100
GfBV-seg22-PTP70
GiBV-seg25-PTP120
GfBV-seg25-PTP70
GiBV-seg25-PTP100
GfBV-seg25-PTP50
GfBV-seg20-PTP280
GiBV-seg20-PTP120
GiBV-seg20-PTP100
GfBV-seg20-PTP300
CcBV_PTPP
CvBV_PTP4
Pvest
Psesa
Prube
Pmargi
Pglom
Pmela
CcBV_PTPL
CvBV_PTP6
Lrube
CcBV_PTPK
Ksesa
Kchilo
Krube
CcBV_PTPQ
CvBV_PTP3
Qrube
Qvest
GiBV-seg25-PTP130
GiBV-seg25-PTP80
CvBV_PTP9
CgBV_PTP9
GfBV-seg20-PTP260
GiBV-seg20-PTP140
GiBV-seg25-PTP70
CcBV_PTPE
Erube
Emargi
Xrube
CcBV_PTPX
CvBV_PTP33
CvBV_PTP20
Xmela
EX3chilo
EX5chilo
EX10chilo
EX1chilo
EX4chilo
EX8chilo
EX2chilo
EX7chilo
EX9chilo
EX6chilo
EX4mela
EX5mela
EX6mela
EX8mela
EX10mela
EX1vest
EX2vest
EX8vest
EX10vest
EX12vest
CsmBV_PTPEX
CskBV_PTPEX1
CskBV_PTPEX2
GfBV-seg22-PTP80
GiBV-seg22-PTP110
GiBV-seg23-PTP150
CcBV_PTPD
CvBV_PTP2
CcBV_PTPM
CvBV_PTP5
Mchilo
Msesa
Mflavi
GiBV-seg25-PTP110
GfBV-seg25-PTP60
GfBV-seg20-PTP290
GiBV-seg20-PTP110
CcBV_PTPS
CvBV_PTP32
CvBV_PTP19
CskBV_PTPS
GiBV-seg22-PTP120
GiBV-seg23-PTP140
GfBV-seg22-PTP90
GfBV-seg23-PTP100
CcBV_PTPH
CvBV_PTP29
Hglom
Hrube
Hchilo
Hflavi
GiBV-seg28-PTP40
GfBV-seg28-PTP40
CcBV_PTPB

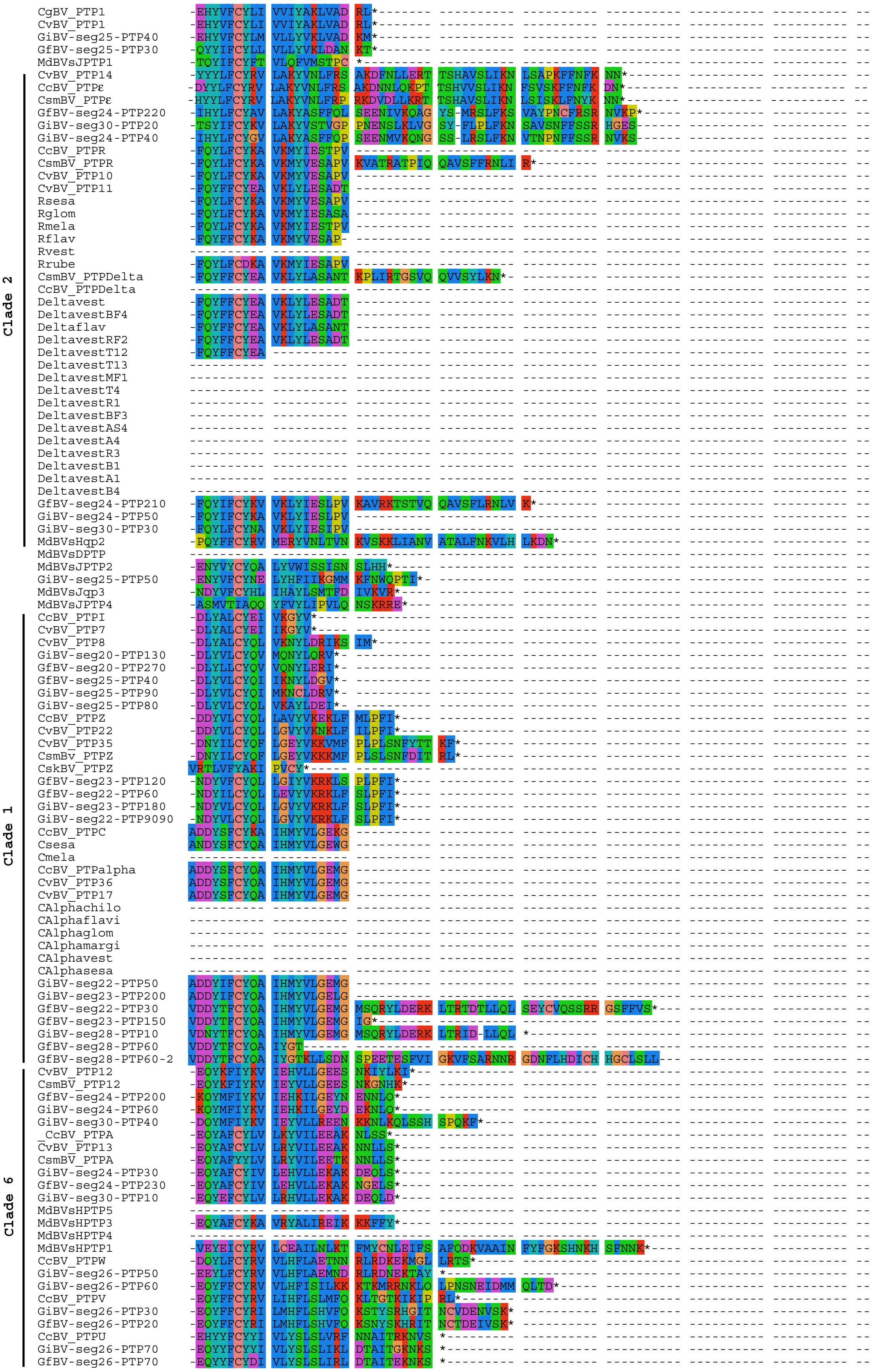
Clade 2
Clade 1
Clade 6
CgBV_PTP1
CvBV_PTP1
GiBV-seg25-PTP40
GfBV-seg25-PTP30
MdBVsJPTP1
CvBV_PTP14
CcBV_PTPε
CsmBV_PTPε
GfBV-seg24-PTP220
GiBV-seg30-PTP20
GiBV-seg24-PTP40
CcBV_PTPR
CsmBV_PTPR
CvBV_PTP10
CvBV_PTP11
Rsesa
Rglom
Rmela
Rflav
Rvest
Rrube
CsmBV_PTPDelta
CcBV_PTPDelta
Deltavest
DeltavestBF4
Deltaflav
DeltavestRF2
DeltavestT12
DeltavestT13
DeltavestMF1
DeltavestT4
DeltavestR1
DeltavestBF3
DeltavestAS4
DeltavestA4
DeltavestR3
DeltavestB1
DeltavestA1
DeltavestB4
GfBV-seg24-PTP210
GiBV-seg24-PTP50
GiBV-seg30-PTP30
MdBVsHqp2
MdBVsDPTP
MdBVsJPTP2
GiBV-seg25-PTP50
MdBVsJqp3
MdBVsJPTP4
CcBV_PTPI
CvBV_PTP7
CvBV_PTP8
GiBV-seg20-PTP130
GfBV-seg20-PTP270
GfBV-seg25-PTP40
GiBV-seg25-PTP90
GiBV-seg25-PTP80
CcBV_PTPZ
CvBV_PTP22
CvBV_PTP35
CsmBv_PTPZ
CskBV_PTPZ
GfBV-seg23-PTP120
GfBV-seg22-PTP60
GiBV-seg23-PTP180
GiBV-seg22-PTP9090
CcBV_PTPC
Csesa
Cmela
CcBV_PTPalpha
CvBV_PTP36
CvBV_PTP17
CAlphachilo
CAlphaflavi
CAlphaglom
CAlphamargi
CAlphavest
CAlphasesa
GiBV-seg22-PTP50
GiBV-seg23-PTP200
GfBV-seg22-PTP30
GfBV-seg23-PTP150
GiBV-seg28-PTP10
GfBV-seg28-PTP60
GfBV-seg28-PTP60-2
CvBV_PTP12
CsmBV_PTP12
GfBV-seg24-PTP200
GiBV-seg24-PTP60
GiBV-seg30-PTP40
_CcBV_PTPA
CvBV_PTP13
CsmBV_PTPA
GiBV-seg24-PTP30
GfBV-seg24-PTP230
GiBV-seg30-PTP10
MdBVsHPTP5
MdBVsHPTP3
MdBVsHPTP4
MdBVsHPTP1
CcBV_PTPW
GiBV-seg26-PTP50
GiBV-seg26-PTP60
CcBV_PTPV
GiBV-seg26-PTP30
GfBV-seg26-PTP20
CcBV_PTPU
GiBV-seg26-PTP70
GfBV-seg26-PTP70
