# Supplementary material for: Evolutionary mechanisms driving the evolution of a large polydnavirus gene family coding for protein tyrosine phosphatases
Source: BMC Evol Biol. 2012 Dec 27;12:253. doi: 10.1186/1471-2148-12-253 (PMC3573978; doi:10.1186/1471-2148-12-253)
Supplement: Additional file 6 — PTP accession numbers. [file 1471-2148-12-253-S6.doc]

Accession numbers of sequences isolated

| *Bracovirus species* | PTP genes | PTP sequence names | Accession number |
| --- | --- | --- | --- |
| *Cotesia sesamiae Bracovirus* | PTP R | Rsesa | FM864228 |
| *Cotesia glomerata Bracovirus* | PTP R | Rglom | FM864229 |
| *Cotesia melanoscela Bracovirus* | PTP R | Rmela | FM864230 |
| *Cotesia flavipes Bracovirus* | PTP R | Rflavi | FM864231 |
| *Cotesia vestalis Bracovirus* | PTP R | Rvest | FM864232 |
| *Cotesia rubecula Bracovirus* | PTP R | Rrube | FM864233 |
| *Cotesia vestalis Bracovirus* | PTP Δ | Δvest | FM864234 |
| *Cotesia vestalis Bracovirus* | PTP Δ | ΔvestBF4 | FM864235 |
| *Cotesia vestalis Bracovirus* | PTP Δ | Δflavi | FM864236 |
| *Cotesia vestalis Bracovirus* | PTP Δ | ΔvestRF2 | FM864237 |
| *Cotesia vestalis Bracovirus* | PTP Δ | ΔvestT12 | FM864238 |
| *Cotesia vestalis Bracovirus* | PTP Δ | ΔvestT13 | FM864239 |
| *Cotesia vestalis Bracovirus* | PTP Δ | ΔvestMF1 | FM864240 |
| *Cotesia vestalis Bracovirus* | PTP Δ | ΔvestT4 | FM864241 |
| *Cotesia vestalis Bracovirus* | PTP Δ | ΔvestR1 | FM864242 |
| *Cotesia vestalis Bracovirus* | PTP Δ | ΔvestBF3 | FM864243 |
| *Cotesia vestalis Bracovirus* | PTP Δ | ΔvestAS4 | FM864244 |
| *Cotesia vestalis Bracovirus* | PTP Δ | ΔvestA4 | FM864245 |
| *Cotesia vestalis Bracovirus* | PTP Δ | ΔvestR3 | FM864246 |
| *Cotesia vestalis Bracovirus* | PTP Δ | ΔvestB1 | FM864247 |
| *Cotesia vestalis Bracovirus* | PTP Δ | ΔvestA1 | FM864248 |
| *Cotesia vestalis Bracovirus* | PTP Δ | ΔvestAS1 | FM864249 |
| *Cotesia vestalis Bracovirus* | PTP Δ | ΔvestB4 | FM864250 |
| *Cotesia rubecula Bracovirus* | PTP Y | Yrube | FM864251 |
| *Cotesia marginiventris Bracovirus* | PTP Y | Ymargi | FM864252 |
| *Cotesia vestalis Bracovirus* | PTP Y | Yvest | FM864253 |
| *Cotesia sesamiae Bracovirus* | PTP Y | Ysesa | FM864254 |
| *Cotesia flavipes Bracovirus* | PTP Y | Yflavi | FM864255 |
| *Cotesia chilonis Bracovirus* | PTP Y | Ychilo | FM864256 |
| *Cotesia melanoscela Bracovirus* | PTP Y | Ymela | FM864257 |
| *Cotesia sesamiae Bracovirus* | PTP K | Ksesa | FM864258 |
| *Cotesia chilonis Bracovirus* | PTP K | Kchilo | FM864259 |
| *Cotesia rubecula Bracovirus* | PTP K | Krube | FM864260 |
| *Cotesia rubecula Bracovirus* | PTP L | Lrube | FM864261 |
| *Cotesia vestalis Bracovirus* | PTP Q | Qvest | FM864262 |
| *Cotesia rubecula Bracovirus* | PTP Q | Qrube | FM864263 |
| *Cotesia sesamiae Bracovirus* | PTP P | Psesa | FM864264 |
| *Cotesia rubecula Bracovirus* | PTP P | Prube | FM864265 |
| *Cotesia marginiventris Bracovirus* | PTP P | Pmargi | FM864266 |
| *Cotesia glomerata Bracovirus* | PTP P | Pglom | FM864267 |
| *Cotesia melanoscela Bracovirus* | PTP P | Pmela | FM864268 |
| *Cotesia vestalis Bracovirus* | PTP P | Pvest | FM864269 |
| *Cotesia sesamiae Bracovirus* | PTP Cα | Csesa | FM864270 |
| *Cotesia melanoscela Bracovirus* | PTP Cα | Cmela | FM864271 |
| *Cotesia chilonis Bracovirus* | PTP Cα | Cαchilo | FM864272 |
| *Cotesia flavipes Bracovirus* | PTP Cα | Cαflavi | FM864273 |
| *Cotesia glomerata Bracovirus* | PTP Cα | Cαglom | FM864274 |
| *Cotesia marginiventris Bracovirus* | PTP Cα | Cαmargi | FM864275 |
| *Cotesia vestalis Bracovirus* | PTP Cα | Cαvest | FM864276 |
| *Cotesia sesamiae Bracovirus* | PTP Cα | Cαsesa | FM864277 |
| *Cotesia glomerata Bracovirus* | PTP H | Hglom | FM864278 |
| *Cotesia rubecula Bracovirus* | PTP H | Hrube | FM864279 |
| *Cotesia chilonis Bracovirus* | PTP H | Hchilo | FM864280 |
| *Cotesia flavipes Bracovirus* | PTP H | Hflavi | FM864281 |
| *Cotesia chilonis Bracovirus* | PTP M | Mchilo | FM864282 |
| *Cotesia sesamiae Bracovirus* | PTP M | Msesa | FM864283 |
| *Cotesia flavipes Bracovirus* | PTP M | Mflavi | FM864284 |
| *Cotesia vestalis Bracovirus* | PTP M | Mvest2 | FM864285 |
| *Cotesia rubecula Bracovirus* | PTP EX | Erube | FM864287 |
| *Cotesia marginiventris Bracovirus* | PTP EX | Emargi | FM864288 |
| *Cotesia rubecula Bracovirus* | PTP EX | Xrube | FM864290 |
| *Cotesia melanoscela Bracovirus* | PTP EX | Xmela | FM864292 |
| *Cotesia chilonis Bracovirus* | PTP EX | EX3chilo | FM864293 |
| *Cotesia chilonis Bracovirus* | PTP EX | EX5chilo | FM864294 |
| *Cotesia chilonis Bracovirus* | PTP EX | EX10chilo | FM864295 |
| *Cotesia chilonis Bracovirus* | PTP EX | EX1chilo | FM864296 |
| *Cotesia chilonis Bracovirus* | PTP EX | EX4chilo | FM864297 |
| *Cotesia chilonis Bracovirus* | PTP EX | EX8chilo | FM864298 |
| *Cotesia chilonis Bracovirus* | PTP EX | EX2chilo | FM864299 |
| *Cotesia chilonis Bracovirus* | PTP EX | EX7chilo | FM864300 |
| *Cotesia chilonis Bracovirus* | PTP EX | EX9chilo | FM864301 |
| *Cotesia chilonis Bracovirus* | PTP EX | EX6chilo | FM864302 |
| *Cotesia melanoscela Bracovirus* | PTP EX | EX4mela | FM864303 |
| *Cotesia melanoscela Bracovirus* | PTP EX | EX5mela | FM864304 |
| *Cotesia melanoscela Bracovirus* | PTP EX | EX6mela | FM864305 |
| *Cotesia melanoscela Bracovirus* | PTP EX | EX8mela | FM864306 |
| *Cotesia melanoscela Bracovirus* | PTP EX | EX10mela | FM864307 |
| *Cotesia vestalis Bracovirus* | PTP EX | EX1vest | FM864308 |
| *Cotesia vestalis Bracovirus* | PTP EX | EX2vest | FM864309 |
| *Cotesia vestalis Bracovirus* | PTP EX | EX8vest | FM864310 |
| *Cotesia vestalis Bracovirus* | PTP EX | EX10vest | FM864311 |
| *Cotesia vestalis Bracovirus* | PTP EX | EX12vest | FM864312 |

Accession numbers of sequences used (Espagne et al. 2004 ; Gundersen-Rindal and Pedroni 2006 ; Webb et al. 2006; Desjardins et al. 2008, Chen et al. 2011)

| *Bracovirus species* | PTP genes | PTP sequence names | Accession number |
| --- | --- | --- | --- |
| ***Cotesia congregata Bracovirus*** | PTP B | CcBV PTPB (segment 1) | CAG17379 |
| *Cotesia congregata Bracovirus* | PTP I | CcBV PTPI (segment1) | CAG17380 |
| *Cotesia congregata Bracovirus* | PTP K | CcBV PTPK (segment1) | CAG17384 |
| *Cotesia congregata Bracovirus* | PTP L | CcBV PTPL (segment1) | CAG17385 |
| *Cotesia congregata Bracovirus* | PTP M | CcBV PTPM (segment1) | CAG17386 |
| *Cotesia congregata Bracovirus* | PTP P | CcBV PTPP (segment1) | CAG17387 |
| *Cotesia congregata Bracovirus* | PTP Q | CcBV PTPQ (segment1) | CAG17388 |
| *Cotesia congregata Bracovirus* | PTP D | CcBV PTPD (segment 1) | CAG17389 |
| *Cotesia congregata Bracovirus* | PTP H | CcBV PTPH (segment4) | CAG17404 |
| *Cotesia congregata Bracovirus* | PTP O | CcBV PTPO (segment4) | CAG17405 |
| *Cotesia congregata Bracovirus* | PTP R | CcBV PTPR( segment7) | CAG17418 |
| *Cotesia congregata Bracovirus* | PTP E | CcBV PTPE (segment10) | CAG17426 |
| *Cotesia congregata Bracovirus* | PTP S | CcBV PTPS (segment10) | CAG17427 |
| *Cotesia congregata Bracovirus* | PTP T | CcBV PTPT (segment 10) | CAG17428 |
| *Cotesia congregata Bracovirus* | PTP C | CcBV PTPC (segment10) | CAG17429 |
| *Cotesia congregata Bracovirus* | PTP N | CcBV PTPN (segment 10) | CAG17431 |
| *Cotesia congregata Bracovirus* | PTP U | CcBV PTPU (segment 14) | CAG17444 |
| *Cotesia congregata Bracovirus* | PTP V | CcBV PTPV (segment 14) | CAG17447 |
| *Cotesia congregata Bracovirus* | PTP W | CcBV PTPW (segment 14) | CAG17449 |
| *Cotesia congregata Bracovirus* | PTP X | CcBV PTPX (segment17) | CAG17455 |
| *Cotesia congregata Bracovirus* | PTP Y | CcBV PTPY (segment17) | CAG17456 |
| *Cotesia congregata Bracovirus* | PTP Z | CcBV PTPZ (segment 17) | CAG17457 |
| *Cotesia congregata Bracovirus* | PTP α | CcBV PTPα (segment17) | CAG17458 |
| *Cotesia congregata Bracovirus* | PTP γ | CcBV PTPγ (segment 17) | AJ640111 |
| *Cotesia congregata Bracovirus* | PTP Δ | CcBV PTPΔ (segment26) | CAG17491 |
| *Cotesia congregata Bracovirus* | PTP A | CcBV PTPA (segment 26) | CAG17496 |
| *Cotesia congregata Bracovirus* | PTP ε | CcBV PTPε (segment 26) | CAG17497 |
| ***Cotesia vestalis Bracovirus*** | PTP 1 | CvBV PTP1 (PTP B) | AAV98015 |
| *Cotesia vestalis Bracovirus* | PTP 2 | CvBV PTP2 (PTP D) | AAV98017 |
| *Cotesia vestalis Bracovirus* | PTP 3 | CvBV PTP3 (PTP Q) | AAV98018 |
| *Cotesia vestalis Bracovirus* | PTP 4 | CvBV PTP4 (PTP P) | AAV98019 |
| *Cotesia vestalis Bracovirus* | PTP 5 | CvBV PTP5 (PTP M) | AAV98020 |
| *Cotesia vestalis Bracovirus* | PTP 6 | CvBV PTP6 (PTP L) | AAV98021 |
| *Cotesia vestalis Bracovirus* | PTP 7 | CvBV PTP7 (PTP I) | AAV98023 |
| *Cotesia vestalis Bracovirus* | PTP 8 | CvBV PTP8 (PTP I) | AAV98024 |
| *Cotesia vestalis Bracovirus* | PTP 9 | CvBV PTP9 (PTP 9) | AAV98025 |
| *Cotesia vestalis Bracovirus* | PTP 10 | CvBV PTP10 (PTP R) | AAV98009 |
| *Cotesia vestalis Bracovirus* | PTP 11 | CvBV PTP11 (PTP Δ) | AAZ04263 |
| *Cotesia vestalis Bracovirus* | PTP 12 | CvBV PTP12 (PTP 12) | AAZ04264 |
| *Cotesia vestalis Bracovirus* | PTP 13 | CvBV PTP13 (PTP A) | AAZ04267 |
| *Cotesia vestalis Bracovirus* | PTP 14 | CvBV PTP14 (PTP ε) | AAZ04268 |
| *Cotesia vestalis Bracovirus* | PTP 17 | CvBV PTP17 (PTP α) | ABK63329 |
| *Cotesia vestalis Bracovirus* | PTP 18 | CvBV PTP18 (PTP γ) | ABK63331 |
| *Cotesia vestalis Bracovirus* | PTP 19 | CvBV PTP19 (PTP S) | ABK63332 |
| *Cotesia vestalis Bracovirus* | PTP 20 | CvBV PTP20 (PTP X) | ABK63333 |
| *Cotesia vestalis Bracovirus* | PTP 21 | CvBV PTP21 (PTP Y) | ABK63334 |
| *Cotesia vestalis Bracovirus* | PTP 22 | CvBV PTP22 (PTP Z) | ABK63335 |
| *Cotesia vestalis Bracovirus* | PTP 29 | CvBV PTP29 (PTP H) | ABK63362 |
| *Cotesia vestalis Bracovirus* | PTP 30 | CvBV PTP30 (PTP O) | ABK63363 |
| *Cotesia vestalis Bracovirus* | PTP 31 | CvBV PTP31 (PTP T) | ABK63317 |
| *Cotesia vestalis Bracovirus* | PTP 32 | CvBV PTP32 (PTP S) | ABK63318 |
| *Cotesia vestalis Bracovirus* | PTP 33 | CvBV PTP33 (PTP E) | ABK63319 |
| *Cotesia vestalis Bracovirus* | PTP 34 | CvBV PTP34 (PTP N) | ABK63321 |
| *Cotesia vestalis Bracovirus* | PTP 35 | CvBV PTP35 (PTP Z) | ABK63322 |
| *Cotesia vestalis Bracovirus* | PTP 36 | CvBV PTP36 (PTP C) | ABK63324 |
| ***Cotesia sesamiae kitale Bracovirus*** | PTP EX | CskBV PTPEX1 | EF710630 |
| *Cotesia sesamiae kitale Bracovirus* | PTP EX | CskBV PTPEX2 | EF710632 |
| *Cotesia sesamiae kitale Bracovirus* | PTP N | CskBV PTPN | EF710632 |
| *Cotesia sesamiae kitale Bracovirus* | PTP S | CskBV PTPS | EF710632 |
| *Cotesia sesamiae kitale Bracovirus* | PTP T | CskBV PTPT | EF710632 |
| *Cotesia sesamiae kitale Bracovirus* | PTP Z | CskBV PTPZ | EF710632 |
| ***Cotesia sesamiae mombasa Bracovirus*** | PTP A | CsmBV PTPA | EF710640 |
| *Cotesia sesamiae mombasa Bracovirus* | PTP EX | CsmBV PTPEX | EF710639 |
| *Cotesia sesamiae mombasa Bracovirus* | PTP N | CsmBV PTPN | EF710639 |
| *Cotesia sesamiae mombasa Bracovirus* | PTP Z | CsmBV PTPZ | EF710639 |
| *Cotesia sesamiae mombasa Bracovirus* | PTP T | CsmBV PTPT | EF710639 |
| *Cotesia sesamiae mombasa Bracovirus* | PTP R | CsmBV PTPR | EF710636 |
| *Cotesia sesamiae mombasa Bracovirus* | PTP Δ | CsmBV PTPΔ | EF710640 |
| *Cotesia sesamiae mombasa Bracovirus* | PTP ε | CsmBV PTPε | EF710640 |
| *Cotesia sesamiae mombasa Bracovirus* | PTP 12 | CsmBV PTP12 | EF710640 |
| ***Cotesia glomerata Bracovirus*** | PTP 9 | CgBV PTP9 | AAR29979 |
| *Cotesia glomerata Bracovirus* | PTP B | CgBV PTP1 | ABH10012 |
| ***Glyptapanteles indiensis Bracovirus*** | PTP 100 | GiBV-seg20-PTP100 (Glypta PTP) | ACE75455 |
| *Glyptapanteles indiensis Bracovirus* | PTP 110 | GiBV-seg20-PTP110 (PTP M) | ACE75456 |
| *Glyptapanteles indiensis Bracovirus* | PTP 120 | GiBV-seg20-PTP120 (Glypta PTP) | ACE75457 |
| *Glyptapanteles indiensis Bracovirus* | PTP 130 | GiBV-seg20-PTP130 (PTP I) | ACE75458 |
| *Glyptapanteles indiensis Bracovirus* | PTP 140 | GiBV-seg20-PTP140 (PTP 9) | ACE75459 |
| *Glyptapanteles indiensis Bracovirus* | PTP 50 | GiBV-seg22-PTP50 (PTP α) | ACE75302 |
| *Glyptapanteles indiensis Bracovirus* | PTP 90 | GiBV-seg22-PTP90 (PTP Z) | ACE75306 |
| *Glyptapanteles indiensis Bracovirus* | PTP 100 | GiBV-seg22-PTP100 (PTP Y) | ACE75307 |
| *Glyptapanteles indiensis Bracovirus* | PTP 110 | GiBV-seg22-PTP110 (PTP X) | ACE75308 |
| *Glyptapanteles indiensis Bracovirus* | PTP 120 | GiBV-seg22-PTP120 (PTP S) | ACE75309 |
| *Glyptapanteles indiensis Bracovirus* | PTP 130 | GiBV-seg22-PTP130 (PTP γ) | ACE75310 |
| *Glyptapanteles indiensis Bracovirus* | PTP 140 | GiBV-seg23-PTP140 (PTP S) | ACE75311 |
| *Glyptapanteles indiensis Bracovirus* | PTP 150 | GiBV-seg23-PTP150 (PTP E) | ACE75312 |
| *Glyptapanteles indiensis Bracovirus* | PTP 160 | GiBV-seg23-PTP160 (PTP Y) | ACE75313 |
| *Glyptapanteles indiensis Bracovirus* | PTP 170 | GiBV-seg23-PTP170 (PTP N) | ACE75314 |
| *Glyptapanteles indiensis Bracovirus* | PTP 180 | GiBV-seg23-PTP180 (PTP Z) | ACE75315 |
| *Glyptapanteles indiensis Bracovirus* | PTP 200 | GiBV-seg23-PTP200 (PTP C) | ACE75317 |
| *Glyptapanteles indiensis Bracovirus* | PTP 30 | GiBV-seg24-PTP30 (PTP A) | ACE75321 |
| *Glyptapanteles indiensis Bracovirus* | PTP 40 | GiBV-seg24-PTP40 (PTP ε) | ACE75322 |
| *Glyptapanteles indiensis Bracovirus* | PTP 50 | GiBV-seg24-PTP50 (PTP Δ) | ACE75323 |
| *Glyptapanteles indiensis Bracovirus* | PTP 60 | GiBV-seg24-PTP60 (PTP 12) | ACE75324 |
| *Glyptapanteles indiensis Bracovirus* | PTP 40 | GiBV-seg25-PTP40 (PTP B) | ACE75364 |
| *Glyptapanteles indiensis Bracovirus* | PTP 50 | GiBV-seg25-PTP50 (Glypta PTP) | ACE75365 |
| *Glyptapanteles indiensis Bracovirus* | PTP 70 | GiBV-seg25-PTP70 (PTP 9) | ACE75367 |
| *Glyptapanteles indiensis Bracovirus* | PTP 80 | GiBV-seg25-PTP80 (PTP I) | ACE75368 |
| *Glyptapanteles indiensis Bracovirus* | PTP 90 | GiBV-seg25-PTP90 (PTP I) | ACE75369 |
| *Glyptapanteles indiensis Bracovirus* | PTP 100 | GiBV-seg25-PTP100 (Glypta PTP) | ACE75370 |
| *Glyptapanteles indiensis Bracovirus* | PTP 110 | GiBV-seg25-PTP110 (PTP M) | ACE75371 |
| *Glyptapanteles indiensis Bracovirus* | PTP 120 | GiBV-seg25-PTP120 (Glypta PTP) | ACE75372 |
| *Glyptapanteles indiensis Bracovirus* | PTP 130 | GiBV-seg25-PTP130 (PTP Q) | ACE75373 |
| *Glyptapanteles indiensis Bracovirus* | PTP 30 | GiBV-seg26-PTP30 (PTP V) | ACE75352 |
| *Glyptapanteles indiensis Bracovirus* | PTP 50 | GiBV-seg26-PTP50 (PTP W) | ACE75354 |
| *Glyptapanteles indiensis Bracovirus* | PTP 60 | GiBV-seg26-PTP60 (PTP W) | ACE75355 |
| *Glyptapanteles indiensis Bracovirus* | PTP 70 | GiBV-seg26-PTP70 (PTP U) | ACE75356 |
| *Glyptapanteles indiensis Bracovirus* | PTP 70 | GiBV-seg26-PTP70 (PTP U) | ACE75356 |
| *Glyptapanteles indiensis Bracovirus* | PTP 10 | GiBV-seg28-PTP10 (PTP Cα) | ACE75472 |
| *Glyptapanteles indiensis Bracovirus* | PTP 40 | GiBV-seg28-PTP40 (PTP H) | ACE75475 |
| *Glyptapanteles indiensis Bracovirus* | PTP 50 | GiBV-seg28-PTP50 (PTP O) | ACE75476 |
| *Glyptapanteles indiensis Bracovirus* | PTP 60 | GiBV-seg28-PTP60 (PTP O) | ACE75477 |
| *Glyptapanteles indiensis Bracovirus* | PTP 10 | GiBV-seg30-PTP10 (PTP A) | ACE75482 |
| *Glyptapanteles indiensis Bracovirus* | PTP 30 | GiBV-seg30-PTP30 (PTP R) | ACE75484 |
| *Glyptapanteles indiensis Bracovirus* | PTP 40 | GiBV-seg30-PTP40 (PTP 12) | ACE75485 |
| ***Glyptapanteles flavicoxis Bracovirus*** | PTP 260 | GfBV-seg20-PTP260 (PTP 9) | ACE75165 |
| *Glyptapanteles flavicoxis Bracovirus* | PTP 270 | GfBV-seg20-PTP270 (PTP I) | ACE75166 |
| *Glyptapanteles flavicoxis Bracovirus* | PTP 280 | GfBV-seg20-PTP280 (Glypta PTP) | ACE75167 |
| *Glyptapanteles flavicoxis Bracovirus* | PTP 290 | GfBV-seg20-PTP290 (PTP M) | ACE75168 |
| *Glyptapanteles flavicoxis Bracovirus* | PTP 300 | GfBV-seg20-PTP300 (Glypta PTP) | ACE75169 |
| *Glyptapanteles flavicoxis Bracovirus* | PTP 30 | GfBV-seg22-PTP30 (PTP α) | ACE75212 |
| *Glyptapanteles flavicoxis Bracovirus* | PTP 60 | GfBV-seg22-PTP60 (PTP Z) | ACE75215 |
| *Glyptapanteles flavicoxis Bracovirus* | PTP 70 | GfBV-seg22-PTP70 (PTP Y) | ACE75216 |
| *Glyptapanteles flavicoxis Bracovirus* | PTP 80 | GfBV-seg22-PTP80 (PTP X) | ACE75217 |
| *Glyptapanteles flavicoxis Bracovirus* | PTP 90 | GfBV-seg22-PTP90 (PTP S) | ACE75218 |
| *Glyptapanteles flavicoxis Bracovirus* | PTP 100 | GfBV-seg23-PTP100 (PTP S) | ACE75219 |
| *Glyptapanteles flavicoxis Bracovirus* | PTP 110 | GfBV-seg23-PTP110 (PTP Y) | ACE75220 |
| *Glyptapanteles flavicoxis Bracovirus* | PTP 120 | GfBV-seg23-PTP120 (PTP Z) | ACE75221 |
| *Glyptapanteles flavicoxis Bracovirus* | PTP 150 | GfBV-seg23-PTP150 (PTP C) | ACE75224 |
| *Glyptapanteles flavicoxis Bracovirus* | PTP 200 | GfBV-seg24-PTP200 (PTP 12) | ACE75202 |
| *Glyptapanteles flavicoxis Bracovirus* | PTP 220 | GfBV-seg24-PTP220 (PTP ε) | ACE75203 |
| *Glyptapanteles flavicoxis Bracovirus* | PTP 210 | GfBV-seg24-PTP210 (PTP R) | ACE75204 |
| *Glyptapanteles flavicoxis Bracovirus* | PTP 230 | GfBV-seg24-PTP230 (PTP A) | ACE75205 |
| *Glyptapanteles flavicoxis Bracovirus* | PTP 30 | GfBV-seg25-PTP30 (PTP B) | ACE75280 |
| *Glyptapanteles flavicoxis Bracovirus* | PTP 40 | GfBV-seg25-PTP40 (PTP I) | ACE75281 |
| *Glyptapanteles flavicoxis Bracovirus* | PTP 50 | GfBV-seg25-PTP50 (Glypta PTP) | ACE75282 |
| *Glyptapanteles flavicoxis Bracovirus* | PTP 60 | GfBV-seg25-PTP60 (PTP M) | ACE75283 |
| *Glyptapanteles flavicoxis Bracovirus* | PTP 70 | GfBV-seg25-PTP70 (Glypta PTP) | ACE75284 |
| *Glyptapanteles flavicoxis Bracovirus* | PTP 80 | GfBV-seg25-PTP80 (PTP Q) | ACE75285 |
| *Glyptapanteles flavicoxis Bracovirus* | PTP 20 | GfBV-seg26-PTP20 (PTP V) | ACE75503 |
| *Glyptapanteles flavicoxis Bracovirus* | PTP 70 | GfBV-seg26-PTP70 (PTP U) | ACE75504 |
| *Glyptapanteles flavicoxis bracovirus* | PTP 10 | GfBV-seg27-PTP10 (PTP N) | ACE75253 |
| *Glyptapanteles flavicoxis Bracovirus* | PTP 20 | GfBV-seg28-PTP20 (PTP O) | ACE75254 |
| *Glyptapanteles flavicoxis Bracovirus* | PTP 30 | GfBV-seg28-PTP30 (PTP O) | ACE75255 |
| *Glyptapanteles flavicoxis Bracovirus* | PTP 40 | GfBV-seg28-PTP40 (PTP H) | ACE75256 |
| *Glyptapanteles flavicoxis Bracovirus* | PTP 60 | GfBV-seg28-PTP60 (PTP Cα) | ACE75258 |
| *Glyptapanteles flavicoxis Bracovirus* | PTP 60-2 | GfBV-seg28-PTP220 (PTP Cα) | ACE75274 |
| ***Microplitis demolitor Bracovirus*** | PTP | MdBVsNqp3 | AAW51807 |
| *Microplitis demolitor Bracovirus* | PTP | MdBVsJqp3 | AAW51793 |
| *Microplitis demolitor Bracovirus* | PTP | MdBVsHqp2 | AAW51786 |
| *Microplitis demolitor Bracovirus* | PTP | MdBVsHPTP3 | AAW51787 |
| *Microplitis demolitor Bracovirus* | PTP | MdBVsJPTP1 | AAW51795 |
| *Microplitis demolitor Bracovirus* | PTP | MdBVsJPTP4 | AAW51792 |
| *Microplitis demolitor Bracovirus* | PTP | MdBVsJPTP2 | AAW51794 |
| *Microplitis demolitor Bracovirus* | PTP | MdBVsDPTP | AAW51780 |
| *Microplitis demolitor Bracovirus* | PTP | MdBVsHPTP1 | AAW51785 |
| *Microplitis demolitor Bracovirus* | PTP | MdBVsHPTP4 | AAW51788 |
| *Microplitis demolitor Bracovirus* | PTP | MdBVsHPTP5 | AAW51790 |
| *Microplitis demolitor Bracovirus* | PTP | MdBVsNPTP1 | AAW51808 |
| *Microplitis demolitor Bracovirus* | PTP | MdBVsNPTP3 | AAW51803 |
