# Supplementary material for: Evolutionary mechanisms driving the evolution of a large polydnavirus gene family coding for protein tyrosine phosphatases
Source: BMC Evol Biol. 2012 Dec 27;12:253. doi: 10.1186/1471-2148-12-253 (PMC3573978; doi:10.1186/1471-2148-12-253)
Supplement: Additional file 7 — Primers used for PTP amplification in different Cotesia species. [file 1471-2148-12-253-S7.doc]

Primers used for PTP amplification in different *Cotesia* species

| **Name** | **Direction** | **Sequence** |
| --- | --- | --- |
| PTPR5 | Forward | tggatcggactattgatgagc |
| PTPR3 | Reverse | ACAGCTTGTTGGATCGGAGT |
| PTPΔ5 | Forward | gcaaacaaaatggcacatga |
| PTPΔ3 | Reverse | TCGGACCGGACTTGTCTTTA |
| PTPH5 | Forward | CCACATTTTTCAAAGTTGGTGA |
| PTPH3 | Reverse | CTGAACAACAAATCCACGTCA |
| PTPS5 | Forward | TGGCTACCAACCTCTCAATG |
| PTPS3 | Reverse | TCCAGCGACAATAAATACGC |
| PTPM5 | Forward | CCGATTTGTTTGCACCTTTT |
| PTPM3 | Reverse | AAGTGCAACAAAACACTGTGC |
| PTPE5 | Forward | tgagcaagtagccgaatcaag |
| PTPE3 | Reverse | CGATGACAGAATAATCGTTT |
| PTPX5 | Forward | gaagcaagtagctgaatctga |
| PTPX3 | Reverse | CGATTACAGAGAAATCGGAA |
| PTPL5 | Forward | gaatgcaaaaactcgccatt |
| PTPL3 | Reverse | TGCAGGCAATCGTATCTTTG |
| PTPK5 | Forward | ttttctggagacctgggaaa |
| PTPK3 | Reverse | GAACGCGTTAAATAGAAACGAA |
| PTPQ5 | Forward | TGGGTTGTGGCAACTCTAAA |
| PTPQ3 | Reverse | GATATGTCAATGGCGCAGAA |
| PTPP5 | Forward | TTCAAAAACGCTAAGCCGTAA |
| PTPP3 | Reverse | TGCCTTTCCTTTCTTAGATTCG |
| PTPY5 | Forward | TGGGGAGTGGAAATTCTAAGTC |
| PTPY3 | Reverse | TCAACATAACAAAGCAAAACTGC |
| PTPC5 | Forward | cgaagagctatctgccgttg |
| PTPC3 | Reverse | TGTTTTATCGAGTGAGTTCT |
| PTPα5 | Forward | tgagtaccgaattcgaagagc |
| PTPα3 | Reverse | TGTTTTATCAATTGAGTCCC |
